# When the brain goes diving: transcriptome analysis reveals a reduced aerobic energy metabolism and increased stress proteins in the seal brain

**DOI:** 10.1186/s12864-016-2892-y

**Published:** 2016-08-09

**Authors:** Andrej Fabrizius, Mariana Leivas Müller Hoff, Gerhard Engler, Lars P. Folkow, Thorsten Burmester

**Affiliations:** 1 Institute of Zoology, Biocenter Grindel, University of Hamburg, Martin-Luther-King-Platz 3, D-20146 Hamburg, Germany; 2 Department of Neurophysiology and Pathophysiology, University Medical Center Hamburg-Eppendorf, 20246 Hamburg, Germany; 3 Department of Arctic and Marine Biology, University of Tromsø – The Arctic University of Norway, NO-9037 Tromsø, Norway

**Keywords:** Brain, Calcium, Diving, Glucose, Hypoxia, Hooded seal, Marine mammals, Stress

## Abstract

**Background:**

During long dives, the brain of whales and seals experiences a reduced supply of oxygen (hypoxia). The brain neurons of the hooded seal (*Cystophora cristata*) are more tolerant towards low-oxygen conditions than those of mice, and also better survive other hypoxia-related stress conditions like a reduction in glucose supply and high concentrations of lactate. Little is known about the molecular mechanisms that support the hypoxia tolerance of the diving brain.

**Results:**

Here we employed RNA-seq to approach the molecular basis of the unusual stress tolerance of the seal brain. An Illumina-generated transcriptome of the visual cortex of the hooded seal was compared with that of the ferret (*Mustela putorius furo*), which served as a terrestrial relative. Gene ontology analyses showed a significant enrichment of transcripts related to translation and aerobic energy production in the ferret but not in the seal brain. Clusterin, an extracellular chaperone, is the most highly expressed gene in the seal brain and fourfold higher than in the ferret or any other mammalian brain transcriptome. The largest difference was found for S100B, a calcium-binding stress protein with pleiotropic function, which was 38-fold enriched in the seal brain. Notably, significant enrichment of S100B mRNA was also found in the transcriptomes of whale brains, but not in the brains of terrestrial mammals.

**Conclusion:**

Comparative transcriptomics indicates a lower aerobic capacity of the seal brain, which may be interpreted as a general energy saving strategy. Elevated expression of stress-related genes, such as clusterin and S100B, possibly contributes to the remarkable hypoxia tolerance of the brain of the hooded seal. Moreover, high levels of S100B that possibly protect the brain appear to be the result of the convergent adaptation of diving mammals.

**Electronic supplementary material:**

The online version of this article (doi:10.1186/s12864-016-2892-y) contains supplementary material, which is available to authorized users.

## Background

A shortage in the supply of oxygen (hypoxia) usually has a devastating impact on the function and the survival of the mammalian brain. Hypoxia is also involved in and is the cause of many neuronal disorders in humans, for example, Alzheimer’s disease [1], Parkinson’s disease [2] and cerebral ischaemia (stroke) [3–5]. A lack of oxygen usually results in irreversible damage to the brain within few minutes and, eventually, death [4]. By contrast, the brain of diving mammals, i.e. whales and seals, may survive recurrent and extended periods of systemic hypoxia without damage [6–12].

The remarkable dive capacity of many marine mammals is brought about by a combination of various behavioural, anatomical and physiological adaptations [7–10, 13–16]. These adaptations that enhance O_2_ supply comprise high levels of haemoglobin and myoglobin, a large blood volume, increased blood stores, an enhanced capacity for anaerobic metabolism and resource-conserving cardiovascular adjustments such as bradycardia and peripheral vasoconstriction [11, 15, 17, 18]. In addition, marine mammals display particular adaptations to swimming, to coping with exposure to a hyperbaric environment, to thermoregulation in cold water, towards enhanced water conservation, and also show adaptations of the sensory organs [15, 19, 20].

The hooded seal (*C. cristata*) displays an astonishing diving capacity, with a maximum dive duration of close to 1 h and a maximum recorded depth of > 1,000 m [21]. During the dive, the arterial blood *P*O_2_ drops dramatically, as also shown for some other deep-diving species [6, 12, 17, 22], and thus the seal brain has to deal with long periods of reduced O_2_ supply. Electrophysiological studies using brain slices demonstrated that the neurons of the hooded seal remained active under severe hypoxia and persisted up to 1 h while the neurons from mice died after only a few minutes [23, 24]. The brain of the hooded seal tolerates low glucose or high lactate levels, in normoxia as well as under hypoxia [25]. The hypoxia tolerance of the neurons of the hooded seal may be at least partly due to a shift of the oxidative metabolism from the neurons to the astrocytes, as evident from a distinct difference in the distribution of neuroglobin and cytochrome c in the brain of the hooded seal compared to the brains of terrestrial mammals [26, 27].

The adaptation of marine mammals to hypoxia on the genetic level may be partly explained by selective gene duplications and losses [28], or specific substitutions within the coding sequences [29]. However, there is little doubt that changes in the expression of individual genes also contribute to the adaptation. To better understand the molecular basis of the stress tolerance of the hooded seal brain, we have employed an RNA-seq approach and compared the abundances of transcripts in the visual cortex of the hooded seal and the ferret. We found that the brain of the hooded seal expresses fewer genes related to energy metabolism and translation, but showed remarkable levels of two typical stress genes, clusterin and S100B. Notably, a high expression level of S100B was also found in whales, but not in terrestrial mammals.

## Results

### Transcriptomes of the visual cortices of the hooded seal and the ferret

We obtained the transcriptomes from the visual cortex of the hooded seal and the ferret (see Additional file 1: Table S1 and Additional file 2: Table S2), mRNA expression levels were estimated by RNA-seq. A total of 10,298 transcripts had RPKM values >1 in both transcriptomes. For further analyses of differential expression, only transcripts with at least RPKM > 5 in both species were considered, resulting in 6,229 transcripts (Additional file 3: Spreadsheet S1).

The highest mRNA levels in the visual cortex of the hooded seal (Table 1a) were found for the chaperone clusterin (CLU) with 3104.57 RPKM, followed by the neuromodulator prostaglandin D2 synthase (PTGDS; 2213.95 RPKM), the S100 calcium binding protein B (S100B; 1860.29 RPKM), a stress protein with pleiotropic function, the metabolic enzyme glyceraldehyde-3-phosphate dehydrogenase (GAPDH, 1413.17 RPKM) and the hydrolyzing enzyme glycerophosphodiester phosphodiesterase domain containing 2 (GDPD2; 1334.93 RPKM). In the brain of the ferret (Table 1b), the highest levels were found for GAPDH (3728.00 RPKM), the mitochondrially encoded cytochrome C oxidase II (MTCO2; 2266.01 RPKM), a peptidyl-prolyl cis-trans isomerase E-like gene (LOC101679695; 1824.67 RPKM) that catalyzes the isomerization of proline peptide bonds, and calmodulin 1 (1805.51 RPKM) and 2 (1802.73 RPKM), which both mediate the control of various enzymes and other proteins via Ca^2+^.

**Table 1:** Most highly expressed genes in the visual cortex of the hooded seal (A) and ferret (B)

Gene	Gene symbol	Function	Hooded seal (RPKM)	Ferret (RPKM)	Fold difference
A. Hooded seal					
Clusterin	CLU	Chaperone	3104.57	777.62	3.99
Prostaglandin D2 synthase	PTGDS	Neuromodulator	2213.95	1451.49	1.53
S100 calcium binding protein B	S100B	Ca^2+^-binding regulator	1860.29	48.98	37.98
Glyceraldehyde-3-phosphate dehydrogenase	GAPDH	Metabolic enzyme	1413.17	3728.00	−2.64
Glycerophosphodiester phosphodiesterase domain containing 2	GDPD2	Lipid metabolism	1334.93	277.95	4.80
Glutamate-ammonia ligase	GLUL	pH control, removal of ammonia and L-glutamate	1046.64	768.49	1.36
Calmodulin 2	CALM2	Ca^2+^-binding regulator	1002.24	1805.51	−1.80
SPARC-like 1 (hevin)	SPARCL1	Ca^2+^-binding	938.95	536.88	1.75
Aldolase C, fructose-bisphosphate	ALDOC	Metabolic enzyme	918.67	906.26	1.01
Prosaposin	PSAP	Lipid metabolism	883.57	913.66	−1.03
B. Ferret					
Glyceraldehyde-3-phosphate dehydrogenase	GAPDH	Metabolic enzyme	1413.17	3728.00	−2.64
Mitochondrially Encoded Cytochrome C Oxidase II	MTCO2	Respiratory chain	245.64	2266.01	−9.22
Peptidyl-prolyl cis-trans isomerase E-like	LOC101679695	Unknown	98.39	1824.67	−18.55
Calmodulin 2	CALM2	Ca^2+^-binding regulator	1002.24	1805.51	−1.80
Calmodulin 1	CALM1	Ca^2+^-binding regulator	480.64	1802.73	−3.75
Prostaglandin D2 synthase	PTGDS	Neuromodulator	2213.95	1451.49	1.53
Mitochondrial ATP synthase, Beta subunit	ATP5B	Respiratory chain	541.42	1166.96	−2.16
Lactate dehydrogenase B	LDHB	Metabolic enzyme	453.40	1127.78	−2.49
Malate Dehydrogenase 1, NAD	MDH1	Metabolic enzyme	544.42	1055.62	−1.94
Ribosomal protein L26	RPL26	Translation	289.61	1040.08	−3.59

The largest differences between the expression levels in the hooded seal and the ferret brain (Table 2) were found for S100B, which has 37.98 times higher RPKM in the seal brain. The leucine-rich proteoglycan osteoglycin (OGN) was 12.14 times higher in the seal brain, followed by Nei endonuclease VIII-like 2 (NEIL2), a DNA glycosylase involved in the repair of damaged DNA (10.11 × higher in seal brain), the alcohol dehydrogenase hydroxysteroid (17-beta) dehydrogenase 11 (HSD17B11; 6.46 ×), a transcript that encodes a serum protein associated with major histocompatibility complex (MHC) class I proteins (beta-2-microglobulin; B2M; 6.26 ×), cytochrome P450, family 4, subfamily V, polypeptide 2 (CYP4V2; 6.05 ×) that oxidises fatty acids, and the antioxidant gene paraoxonase 2 (PON2).

**Table 2:** Transcripts most highly overrepresented in the seal visual cortex compared to the ferret brain

Differentially expressed gene	Gene symbol	Function	Hooded seal (RPKM)	Ferret (RPKM)	Fold difference
S100 calcium binding protein B	S100B	Ca^2+^-binding regulator	1860.3	49.0	37.98
Osteoglycin	OGN	Growth factor	288.0	23.7	12.14
Nei endonuclease VIII-like 2	NEIL2	DNA repair	51.1	5.1	10.11
Hydroxysteroid (17-beta) dehydrogenase 11	HSD17B11	Steroid synthesis	81.3	12.6	6.46
β-2-microglobulin	B2M	Immune protein	242.1	38.7	6.26
Cytochrome P450, family 4, subfamily V, polypeptide 2	CYP4V2	Lipid metabolism	86.2	14.3	6.05
Paraoxonase 2	PON2	Antioxidant protein	95.9	17.3	5.54
Biglycan	BGN	Development and regeneration	41.1	7.7	5.35
Moesin	MSN	Membrane- cytoskeleton interaction	60.4	11.3	5.33
Transmembrane 4 L six family member 1	TM4SF1	Signal transduction	33.4	6.3	5.31

Only genes with RPKM values > 5 were considered. Putative HLA-DRA genes were excluded

### Functional annotation of transcripts differentially expressed in seal and ferret transcriptomes

**Fig. 1:**
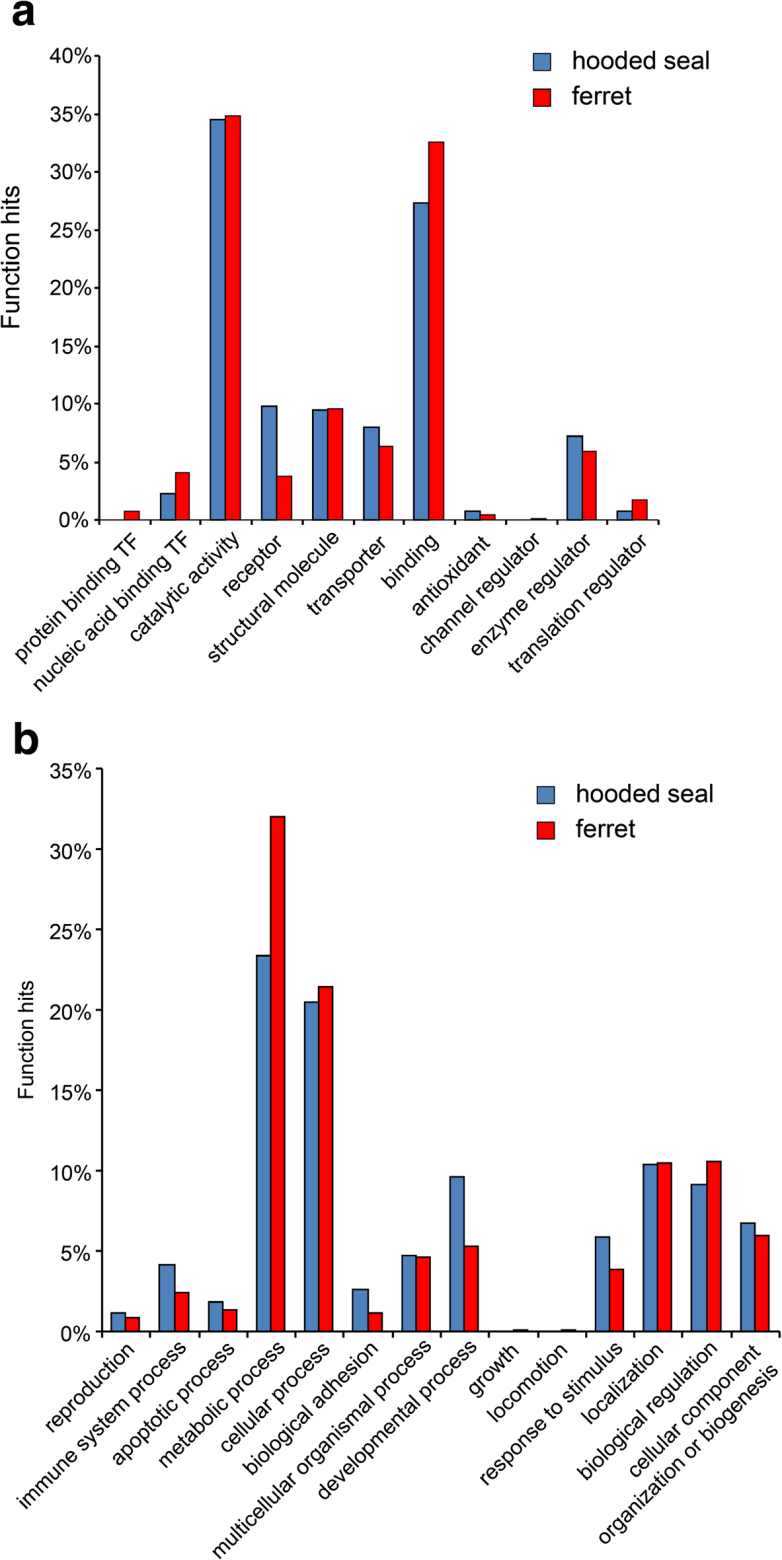
Gene ontology (GO) analyses of the differentially expressed genes in the seal (blue) and ferret (red). Only genes with an at least twofold difference in expression levels and RPKM >5 in both species were considered. **a** GO terms in the PANTHER Annotation Data Set “Molecular Function”. **b** GO terms in the PANTHER Annotation Data Set “Biological Process”

For GO analyses (Fig. 1), only genes with RPKM > 5 in either species (6,229 genes; Additional file 3: Spreadsheet S1) and > twofold expression difference were considered. In the genes (263 with Fold Change > 2) that are more highly expressed in the seal brain, the GO terms “catalytic activity” (91) and “binding” (72) were the highest in the domain “molecular function” (Additional file 4: Table S3). The PANTHER overrepresentation test showed that the enrichment of the GO-Slim terms “structural constituent of cytoskeleton” (2.66-fold; *p =* 0.00644), “oxidoreductase activity” (2.56-fold; *p =* 0.0241) and “catalytic activity” (1.4-fold; *p =* 0.0364) was significant. The terms related to DNA-binding were underrepresented. In the domain “biological process”, the highest numbers were allocated to the GO terms “metabolic process” (115) and “cellular process” (101). A large number of GO terms were found overrepresented, including terms related to glial cells (>5-fold; *p <* 0.05), “oxidation-reduction process” (2.48-fold; *p =* 0.0203) and “response to stress” (1.73-fold; *p =* 0.00221). In the domain “protein class”, the highest numbers were found in the terms “receptor”, “cytoskeletal protein”, “enzyme modulator”, “hydrolase”, “transferase”, “nucleic acid binding”, “transporter”, and “oxidoreductase”. The overrepresentation test showed significant enrichment of terms “actin family cytoskeletal protein” (3.45-fold; *p =* 0.0026) and “oxidoreductase” (2.63-fold; *p =* 0.0263).

We found 1,207 genes that were at least twofold more highly expressed in the brain of the ferret (Additional file 5: Table S4). In the domain “molecular function”, the GO terms “catalytic activity” (374) and “binding” (351) were the highest. Among the GO-Slim terms that showed significant enrichment were “structural constituent of ribosome” (>5-fold; *p =* 1.9 × 10^−22^), “translation initiation factor activity” (2.92-fold; *p =* 0.038) and “oxidoreductase activity” (1.84-fold; *p =* 0.00057). In the domain “biological process”, the highest numbers were allocated to the GO terms “metabolic process” (115) and “cellular process” (101). In the domain “protein class”, the highest numbers were found in the terms “nucleic acid binding” (181), “enzyme modulator” (86) and “transferase” (83). The Overrepresentation Test showed significant enrichment of the terms, among others, test were “oxidative phosphorylation” (4.74-fold; *p =* 0.000265), “respiratory electron transport chain” (2.68-fold; *p =* 0.000128) and “translation” (3.66-fold; *p =* 8.77 × 10^−23^).

### Comparative analysis of mammalian brain transcriptomes

**Fig. 2:**
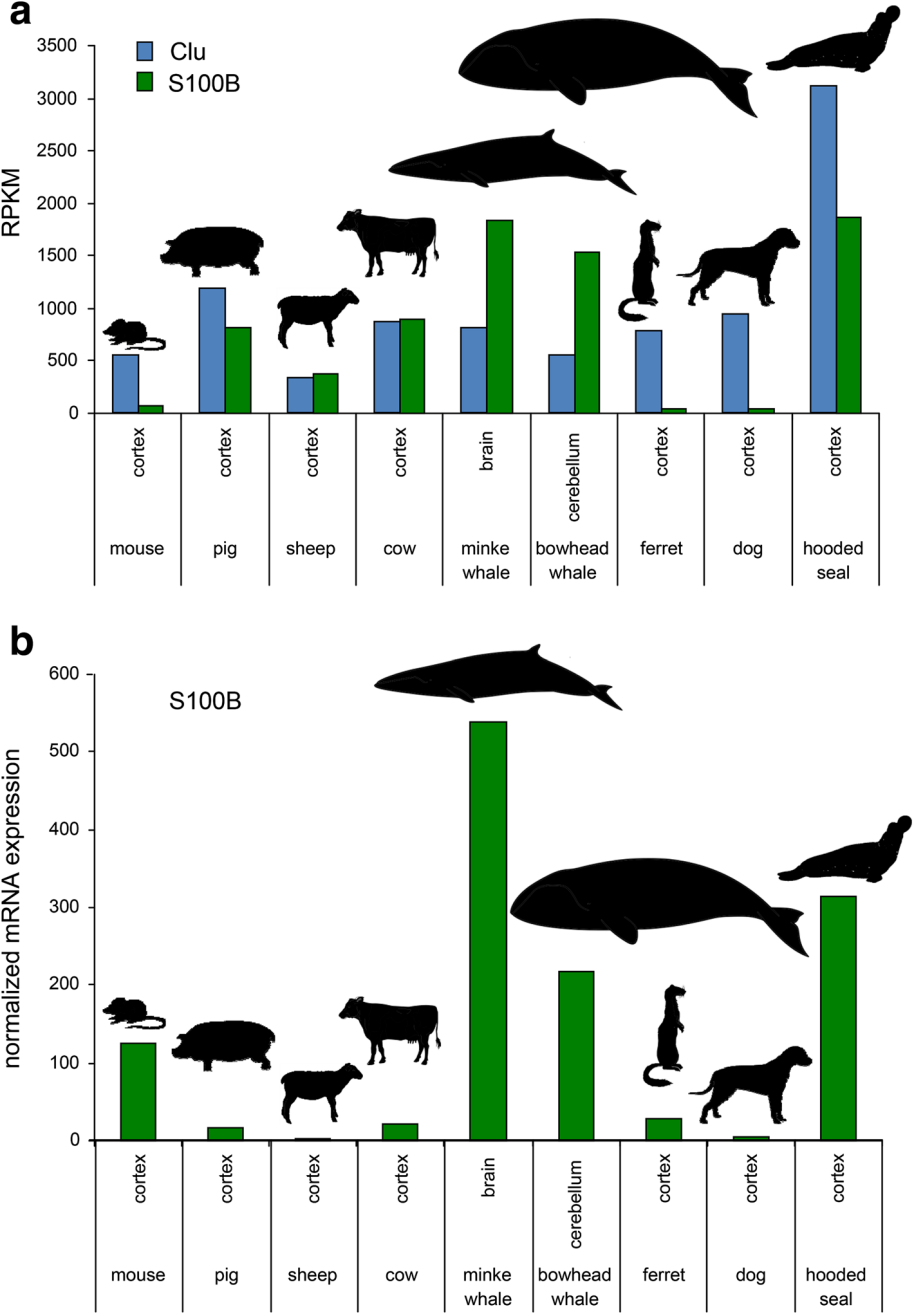
Expression levels of Clu (blue) and S100B (green) in brain tissues of diving and non-diving mammals **a** The specific brain regions and the species are indicated. **b** S100B expression was normalised according to the neuron/glia ratio

The high expression of CLU and S100B in the visual cortex of the hooded seal compared to the ferret cortex was verified by qRT-PCR (Additional file 6: Figure S1). To evaluate the expression levels of CLU and S100B in the brain of other mammals, we obtained the brain transcriptomes of other mammals from the SRA database (Additional file 7: Spreadsheet S2). If available, we selected transcriptomes from the cerebral cortex. For the whale species, only a “brain” transcriptome (without further specification) from the minke whale and a cerebellum transcriptome from the bowhead whale were available. We found high expression of CLU only in the brain transcriptome of the hooded seal but not in any other of the investigated mammalian brain transcriptomes (Fig. 2a). S100B was found to be highly expressed in the hooded seal as well as in the two whale brains. The S100B expression levels were statistically different between diving and non-diving species (two-tailed *t*-test; *p =* 0.0009).

As the neuron/glia ratio may differ between the brain samples and whales may have a higher relative content of glia cells [30], we normalised the S100B expression rates according to the relative expression levels of a neuronal marker (RBFOX3) and a glial marker gene (GFAP) (note: CLU was not normalised because it is expressed in both neurons and glia cells). In fact, the GFAP/RBFOX3 ratios vary between the species up to a factor of >200 (Fig. 2b). However, the normalised expression ratios clearly show significantly higher levels of S100B in the brains of the hooded seal and the two whale species (*p =* 0.0021).

To evaluate the similarities of the brain transcriptomes between different species, correlation coefficients of the transcriptomes were calculated and visualised by a neighbor-joining tree (Additional file 8: Figure S2). No general similarity of the brain transcriptomes of the diving mammals was found. The whale brain transcriptomes cluster with that of the cow and the transcriptomes from the visual cortex of the hooded seal with that of the ferret, probably reflecting the evolutionary relationships of the taxa.

## Discussion

### No indication of higher anaerobic capacity of the seal brain, but evidence for reduced aerobic energy metabolism

At least during long dives, the brain of whales and seals must cope with a reduced supply of oxygen. While the systemic mechanisms that help diving mammals to survive are well studied on the level of the organism [7–10, 13–15, 17, 28, 29], little is known about the molecular mechanisms that help the brain tolerate periods of hypoxia. Notably, the mRNA levels of the key enzymes for anaerobic metabolism, lactate dehydrogenase A and B (LDHA and LDHB), are lower in the visual cortex of the hooded seal than in the ferret (Additional file 3: Spreadsheet S1), confirming qRT-PCR studies and activity tests [31]. Thus, despite the ability of the seal neurons to tolerate extended periods of hypoxia [23–25] and high reported seal brain glycogen levels [6, 25, 32], there is – at least at the transcriptome level – no evidence for a higher anaerobic capacity of the total brain. Rather there appears to exist an adjustment of the labour division between neurons and astrocytes, resulting e.g. in a higher anaerobic capacity of neurons and a higher aerobic metabolism of astrocytes, which may improve neuronal survival [26, 27, 31].

Analysis of the GO terms showed that a large fraction of the genes that are significantly overrepresented in the ferret brain transcriptome are related to the aerobic energy metabolism (e.g., “oxidative phosphorylation”, “respiratory electron transport chain”, “generation of precursor metabolites and energy”) (Additional file 5: Table S4). This is not the case in the seal brain. Thus, rather than having a higher anaerobic capacity, as observed in other hypoxia or anoxia-tolerant species [10, 33, 34], the adaptation of the seal brain to low oxygen conditions may involve a lower aerobic energy metabolism. We also found in the overrepresented ferret genes the enriched GO terms related to translation, which also suggests comparably lower protein synthesis in the seal brain. Because translation is one of the most ATP-demanding cellular processes, its reduction may also contribute to a global energy saving of the seal brain. Similar observations have been made before, e.g. in fish exposed to hypoxia or in anoxic turtles [33, 35, 36].

We further note that we found no evidence that the observed expression patterns are markedly influenced by the use of specific brain areas, sex, or morphological factors like brain or body masses. Correlation analyses of gene expression between the mammalian brain transcriptomes showed a remarkable correlation between gene expression patterns and species phylogeny (Additional file 8: Figure S2). Thus, the phylogenetic relationship appears to be the main driving force of gene expression in the brain, thereby justifying the use of the ferret as close relative for a comparison with the seal.

### Clusterin and S100B may contribute to stress tolerance of the diving brain

CLU (also known as apolipoprotein J) is the most highly expressed gene in the visual cortex of the hooded seal (3104.4 RPKM). The RPKM is much higher than in the visual cortex of the ferret or in other publically available transcriptomes of other mammals (Fig. 2a), suggesting a specific adaptation of the hooded seal brain. The *CLU* gene codes for two distinct products that exert multiple functions [37, 38]. The conventional, secreted form is a heterodimeric glycoprotein that acts as an extracellular chaperone, and it is thought to promote cellular survival. Alternative splicing results in a truncated CLU protein, which interferes with the BAX-mediated apoptosis pathway. In humans, CLU is involved in several neurodegenerative diseases [38]. For example, soluble CLU prevents the aggregation of amyloid β protein in Alzheimer’s disease [39] and high levels of *CLU* mRNA have been associated with aging [40]. Upregulation of CLU has been observed under various stress conditions, including oxidative stress [38]. It is thus conceivable that *CLU* protects the seal brain from the oxidative stress brought about by the hypoxic periods of the dive as well as during reoxygenation when the seal surfaces. In this context, the high levels of Clu mRNA in the seal brain may either be interpreted as a pre-adaptation to the stress conditions during the dives, or Clu expression was induced by the stress conditions during the dives.

The largest difference in expression was found for S100B, which has 38-fold higher RPKM in the hooded seal cortex compared to the ferret cortex (1860.3 vs. 49.0 RPKM). S100B is an EF-hand, Ca^2+^-binding protein that is, at least in the brains of mouse, rat and man, mainly expressed in astrocytes but also in certain neurons [41–43]. We must note that we have no information on the cellular distribution of S100B in the hooded seal, ferret and most other species used in the comparative approach. S100B has intracellular and extracellular functions, and regulates a variety of processes, including cell proliferation, activation of astrocytes during brain damage and disease, as well as promoting cancer. In humans, high levels of S100B have been associated with schizophrenia [44, 45] and other brain-related diseases [46, 47]. Notably, S100B was also found elevated in the brains of the minke and the bowhead whales compared to the brains of non-diving mammals (Fig. 2a), and this difference remains when the RPKM values were corrected according to the glia/neuron ratio (Fig. 2b). Cluster analyses of the correlation coefficients of the expression values of the different transcriptomes exclude that the observed similarities are due to similar brain regions (Additional file 8: Figure S2). Thus, S100B is component of a common stress adaptation mechanism, which must have evolved convergently in whales and seals, and which helps the brain of diving mammals to survive better a reduced oxygen supply. One possible mechanism may be that the high S100B levels enhance the intracellular Ca^2+^ binding sites, thus increasing the Ca^2+^ buffering capacity and thereby reduce hypoxia-induced excitotoxicity, as the massive influx of Ca^2+^ would otherwise cause irreversible neuronal damages and induces apoptosis [3–5]. Alternatively, the high levels of S100B may be an indicator of the brain damage that occurs in response to the stressful life of diving mammals.

Multiple other genes showed remarkable differences in expression levels, but their functions in the brain are more difficult to interpret. For example, previous studies have associated OGN with bone and muscle formation [48] and CYP4V2 is associated in man with retinal dystrophy [49]. However, the 10.1 times higher seal levels of NEIL2, a glycosylase associated with the repair of DNA [50, 51], may be easily explained by increased DNA damage in the hypoxic seal brain. PON2, which metabolizes oxidized arachidonic acid and docosahexaenoic acid [52], is 5.54 times higher in the hooded seal and may be involved in the antioxidative response of the brain cells. However, as for Clu and S100B, it remains unclear, whether genes that were highly expressed in the seal brain reflect an intrinsic feature, or whether these increased in response to the stress evoked by the dives.

## Conclusions

Each year, millions of individuals die or become morbidly ill because of conditions or diseases that reduce oxygen-supply to hypoxia-sensitive tissues such as the brain. Acute metabolic insults like stroke have an especially devastating impact which is mostly impossible to repair. By contrast, brains of diving mammals tolerate extended periods of systemic hypoxia. Differential regulation of specific genes, such as *CLU* and *S100B*, but probably also many others, may be instrumental in the protection of the diving brain. These genes may also be suitable drug targets for drugs that prevent e.g. stroke.

## Methods

### Animals and sample preparation

Adult hooded seals (*n =* 5; female, body mass 142 to 220 kg) were captured in the pack ice of the Greenland Sea, and were euthanised under deep gas anesthesia (ventilation with 1.5–3 % isoflurane [Forene, Abbott, Germany] in air), after initial sedation (intramuscular or intravenous injection of 1.5–3.0 mg zolazepam/tiletamine per kg of body mass). After bleeding and decapitation, brain samples were preserved frozen at−80 °C in RNAlater (Qiagen, Hilden, Germany). Four adult ferrets (*M. putorius furo*) (male; ~2 years old; body mass ~1.5 kg) were obtained from the animal facilities of the University Medical Center Hamburg-Eppendorf (UKE, Germany). The animals were killed in deep anesthesia (Ketamin/Domitor), with an overdose of pentobarbital; subsequently the brain was removed by the veterinarians. Brain samples were preserved at −80 °C in RNAlater.

Total RNA was extracted using peqGOLD Trifast (PEQLAB, Erlangen, Germany) in association with Crystal RNA Mini Kit (Biolab Products, Bebensee, Germany). After quantity and quality analysis using spectrophotometry and gel electrophoresis, RNA samples were used for Illumina sequencing or qRT-PCR.

### Sequencing and assembly of the transcriptomes

A library for paired-end sequencing of 300 nt was generated from 5 μg RNA from the hooded seal visual cortex (adult female). Sequencing was performed with the MiSeq chemistry v3 with an estimated output of 50 million reads (StarSEQ, Mainz, Germany). For the ferret (adult male), a library for paired-end sequencing of 125 nt was generated from 1 μg RNA of the visual cortex and sequencing was performed with the HiSeq2500 chemistry v4 with an estimated output of 25 million reads (GATC Biotech, Konstanz, Germany). Sequence quality analyses were performed via FastQC and CLC-Genomics Workbench (version 7.5). For trimming, all reads with more than two ambiguous characters and with a mean Phred quality of below 15 were discarded. Additionally, the first 14 nucleotides from the 5′ end were trimmed.

For *de novo* assembly of the brain transcriptome of the hooded seal, the quality controlled paired-end reads (12,473,522 reads) were used. After assembly, a backmapping step of the reads was performed. Only contigs with a minimum length of 300 bp were accepted for BLAST analysis. The de novo assembly and backmapping were performed with CLC-Genomics Workbench (version 7.5).

The raw Illumina files of the transcriptomes from the visual cortex of the hooded seal and the ferret are available from the NCBI SRA database under the accession numbers SRR3001184 (Bioproject PRJNA278355) and SRR3000035 (PRJNA305974), respectively.

### Functional transcriptome annotation

To annotate the de novo contigs of the transcriptome of the brain of the hooded seal, a local BLAST search was performed with the BLAST tool of the CLC workbench. Two different protein databases were used to annotate the contigs; firstly, the curated and non-redundant SWISSPROT database and secondly only the human RefSeq protein database (for consequent Gene Ontology (GO) annotation). Only BLAST hits with an expectation value of E < 10^−5^ were accepted. We preferentially annotated ambiguous contigs based on the similarity to walrus (*Odobenus rosmarus*) and ferret (*M. putorius furo*) genes.

To identify overrepresented functional categories among different sets of expressed genes, we used PANTHER (Protein ANalysis THrough Evolutionary Relationships; http://go.pantherdb.org/) Version 10.0 [53]. The GO terms in the domains “molecular function” and “biological process”, and the protein class were reported. Enrichment of categories was evaluated using the PANTHER Overrepresentation Test (release 2015.04.30) using the human genes as reference list. The complete GO terms and PANTHER GO-Slim terms were tested. Categories with *p*-values < 0.05 after Bonferroni correction were considered significant.

### Expression analysis (RNA-seq)

Mapping was performed using the RNA-seq algorithm of the CLC-Genomics Workbench (version 7.5). The ferret Ensembl-build 1.0.75 was used as reference genome. The trimmed seal reads were mapped using the following parameters: 75 % of the read length and 75 % of the nucleotides were required to match the reference for the read to be included in the mapping. The paired read distance was calculated automatically ranging from 145 to 730 bp. Only reads mapping uniquely in the genome were used for RPKM calculation eliminating repetitive sequence bias in read quantification. For the trimmed ferret RNA-seq dataset the parameters were adjusted to 95 % similarity and 95 % length match, for a read to be included in the mapping. The paired read distance was calculated automatically ranging from 90 to 378 bp.

### Comparative transcriptomics

We retrieved the publicly available transcriptomes of mouse (*Mus musculus*) cortex, pig (*Sus scrofa*) cortex, dog (*Canis lupus familiaris*) cortex, cow (*Bos taurus*) cortex, sheep (*Ovis aries*) cortex, minke whale (*Balaenoptera acutorostrata*) brain, and bowhead whale (*Balaena mysticetus*) cerebellum from the SRA database (accession numbers SRX186042, ERX240895, ERX324009, SRX211675, ERX454974, SRX313597, and SRX790347). Each dataset was mapped to the appropriate genome (whale transcriptomes were mapped against the *Bos taurus* genome), and gene expression was calculated as RPKM (Reads Per Kilobase per Million mapped reads). Only genes with RPKM ≥ 5 in the hooded seal were included. Statistical evaluation was performed with GraphPad Prism 6, version 6.01 (La Jolla California USA).

To correct for the different species or brain regions, the glia/neuron ratio of each sample was calculated using the RPKM values of *GFAP* and *RBFOX3* (alias NeuN) as a proxy. Because *RBFOX3* is currently not annotated in the pig genome, we used *SNAP25* as neuronal gene to calculate the glia/neuron ratio in this species. Correlation analysis was done employing the corrected values. The Pearson correlation coefficients were calculated using Microsoft Excel 2013 on 4650 corrected gene expression values. The correlation coefficients of gene expression between species were converted into distances and used to calculate a tree employing the NEIGHBOR program of the PHYLIP 3.68 package.

### Quantitative real-time RT-PCR

First-strand cDNA was synthesised from 1.5 μg total RNA of cortex samples of hooded seal and ferret using the Fermentas RevertAid H^−^ Reverse Transcriptase Kit (Thermo Fisher Scientific, Braunschweig, Germany) according to manufacturer’s instructions. qRT-PCR was performed on the ABI 7500 real-time PCR system with the Power SYBR Green master mix (Applied Biosystems, Darmstadt, Germany) using a 40 cycles protocol (95 °C for 15 s, 60 °C for 15 s, 72 °C for 30 s). Primer sequences are given in Additional file 9: Table S5. The relative mRNA levels were calculated using the cycle threshold (CT) values and were reported as fold changes of the ferret mRNA levels.

## Abbreviations

GO, gene ontology; qRT-PCR, quantitative real-time RT-PCR; RNA-seq, RNA Sequencing; RPKM, reads per kilobase per million mapped reads

## Additional files

Additional file 1: Table S1. Summary of Illumina sequencing. The numbers of reads before and after quality trimming are given. The percentage of reads mapped to the ferret genome is denoted. (PDF 20 kb) Additional file 2: Table S2.
*De novo* assembly of the transcriptome from the seal visual cortex. (PDF 6 kb) Additional file 3: Spreadsheet S1. Comparative gene expression in the seal and ferret brain. Gene expression (RPKM values) in the visual cortex of the hooded seal and the ferret. The cut-off was set to RPKM >5 in both species. (XLSX 406 kb) Additional file 4: Table S3. Genes overrepresented in the seal brain. Ontology analysis of genes that are at least twofold higher expressed in the visual cortex of the hooded seal compared to the ferret visual cortex. A. The GO terms for the domains “molecular function” and “biological process”, and the “protein class” are given. B and C. PANTHER Overrepresentation Test of the domains “molecular function”, “biological process”, and “protein class” using the complete (B) and PANTHER GO-slim terms. (PDF 24 kb) Additional file 5: Table S4. Genes overrepresented in the ferret brain. Ontology analysis of genes that are at least twofold higher expressed in the visual cortex of the ferret compared to the hooded seal visual cortex. A. The GO terms for the domains “molecular function” and “biological process”, and the “protein class” are given. B and C. PANTHER Overrepresentation Test of the domains “molecular function”, “biological process”, and “protein class” using the complete (B) and PANTHER GO-slim terms. (PDF 74 kb) Additional file 6: Figure S1. Comparison of qRT-PCR and RNA-seq results. The changes in selected mRNA levels in visual cortices of the hooded seal and the ferret were estimated by qRT-PCR (black; *n =* 4) and RNA-seq (white). The mRNA levels of the genes *S100B*, *Clu*, *SLC1A6* and *GAPDH* were evaluated. Both methods gave similar results. (PDF 22 kb) Additional file 7: Spreadsheet S2. Gene expression in the mammalian brain. The RPKM values have been calculated for cortices of mouse, pig, dog, cow, sheep, ferret and hooded seal, in the brain of minke whale and the cerebellum of bowhead whale. n.a., not available; n.d., not detected. (XLSX 760 kb) Additional file 8: Figure S2. Correlation of gene expression between mammalian brain transcriptomes. The correlation coefficients were converted into distances and visualised by a neighbor-joining tree. (PDF 27 kb) Additional file 9: Table S5. List of primers. Forward and reverse primers, which had been generated according to the conserved sequences of the ferret and hooded seal genes, used in qRT-PCR expression analyses. (PDF 6 kb)
