# Supplementary material for: When the brain goes diving: transcriptome analysis reveals a reduced aerobic energy metabolism and increased stress proteins in the seal brain
Source: BMC Genomics. 2016 Aug 9;17:583. doi: 10.1186/s12864-016-2892-y (PMC4979143; doi:10.1186/s12864-016-2892-y)
Supplement: Additional file 1: Table S1. — Summary of Illumina sequencing. The numbers of reads before and after quality trimming are given. The percentage of reads mapped to the ferret genome is denoted. (PDF 20 kb) [file 12864_2016_2892_MOESM1_ESM.pdf]

Additional File: Table S1. Summary of Illumina sequencing. The numbers of reads before and after quality trimming are given. The percentage of reads mapped to the ferret genome is denoted.

	**SRA accession number**	**Raw reads**	**Reads after trimming**	**Mapped reads %**
Hooded seal visual cortex	SRR3001184	37,108,070	12,473,522	52.45
Ferret visual cortex	SRR3000035	45,683,734	45,351,462	54.23
