# Supplementary material for: When the brain goes diving: transcriptome analysis reveals a reduced aerobic energy metabolism and increased stress proteins in the seal brain
Source: BMC Genomics. 2016 Aug 9;17:583. doi: 10.1186/s12864-016-2892-y (PMC4979143; doi:10.1186/s12864-016-2892-y)
Supplement: Additional file 2: Table S2. — De novo assembly of the transcriptome from the seal visual cortex. (PDF 6 kb) [file 12864_2016_2892_MOESM2_ESM.pdf]

Additional File 2: Table S2. De novo assembly of the transcriptome from the seal visual cortex.

	**Assembly statistics**
Reads	12,473,522
Contigs	85,821
Singletons	1,919
>1000 nt	23,481
N50	1,470
Max	22,647
Average	1,061
Used for BLASTx (>500 nt)	60,602
blastx Swissprot (E<1e-5)	23,224
blastx human Refseq (E<1e-5)	22,134
