# Supplementary material for: When the brain goes diving: transcriptome analysis reveals a reduced aerobic energy metabolism and increased stress proteins in the seal brain
Source: BMC Genomics. 2016 Aug 9;17:583. doi: 10.1186/s12864-016-2892-y (PMC4979143; doi:10.1186/s12864-016-2892-y)
Supplement: Additional file 4: Table S3. — Genes overrepresented in the seal brain. Ontology analysis of genes that are at least twofold higher expressed in the visual cortex of the hooded seal compared to the ferret visual cortex. A. The GO terms for the domains “molecular function” and “biological process”, and the “protein class” are given. B and C. PANTHER Overrepresentation Test of the domains “molecular function”, “biological process”, and “protein class” using the complete (B) and PANTHER GO-slim terms. (PDF 24 kb) [file 12864_2016_2892_MOESM4_ESM.pdf]

Additional File 4: Table S3. Genes overrepresented in the seal brain. Ontology analysis of genes that are at least twofold higher expressed in the visual cortex of the hooded seal compared to the ferret visual cortex. A. The GO terms for the domains "molecular function" and "biological process", and the "protein class" are given. B and C. PANTHER Overrepresentation Test of the domains "molecular function", "biological process", and "protein class" using the complete (B) and PANTHER GO-slim terms.

**A.**	**Category name**	**Accession**	**number of genes**	**Percent of gene hit against total**	**Function hits**
**Molecular function**					
1	transporter activity	(GO:0005215)	21	8.00%	8.00%
2	translation regulator activity	(GO:0045182)	2	0.80%	0.80%
3	enzyme regulator activity	(GO:0030234)	19	7.20%	7.20%
4	catalytic activity	(GO:0003824)	91	34.60%	34.50%
5	receptor activity	(GO:0004872)	26	9.90%	9.80%
6	nucleic acid binding transcription factor activity	(GO:0001071)	6	2.30%	2.30%
7	antioxidant activity	(GO:0016209)	2	0.80%	0.80%
8	structural molecule activity	(GO:0005198)	25	9.50%	9.50%
9	binding	(GO:0005488)	72	27.40%	27.30%
**Biological process**					
1	cellular component organization or biogenesis	(GO:0071840)	33	12.50%	6.70%
2	cellular process	(GO:0009987)	101	38.40%	20.50%
3	localization	(GO:0051179)	51	19.40%	10.40%
4	apoptotic process	(GO:0006915)	9	3.40%	1.80%
5	reproduction	(GO:0000003)	6	2.30%	1.20%
6	biological regulation	(GO:0065007)	45	17.10%	9.10%
7	response to stimulus	(GO:0050896)	29	11.00%	5.90%
8	developmental process	(GO:0032502)	47	17.90%	9.60%
9	multicellular organismal process	(GO:0032501)	23	8.70%	4.70%
10	biological adhesion	(GO:0022610)	13	4.90%	2.60%
11	metabolic process	(GO:0008152)	115	43.70%	23.40%
12	immune system process	(GO:0002376)	20	7.60%	4.10%
**Protein class**					
1	extracellular matrix protein	(PC00102)	10	3.80%	3.40%
2	protease	(PC00190)	5	1.90%	1.70%
3	cytoskeletal protein	(PC00085)	25	9.50%	8.60%
4	transporter	(PC00227)	20	7.60%	6.90%
5	transferase	(PC00220)	22	8.40%	7.60%

6	oxidoreductase	(PC00176)	20	7.60%	6.90%
7	lyase	(PC00144)	4	1.50%	1.40%
8	cell adhesion molecule	(PC00069)	10	3.80%	3.40%
9	ligase	(PC00142)	1	0.40%	0.30%
10	nucleic acid binding	(PC00171)	22	8.40%	7.60%
11	signaling molecule	(PC00207)	15	5.70%	5.20%
12	enzyme modulator	(PC00095)	24	9.10%	8.30%
13	calcium-binding protein	(PC00060)	5	1.90%	1.70%
14	defense/immunity protein	(PC00090)	12	4.60%	4.10%
15	hydrolase	(PC00121)	23	8.70%	7.90%
16	transfer/carrier protein	(PC00219)	4	1.50%	1.40%
17	membrane traffic protein	(PC00150)	10	3.80%	3.40%
18	phosphatase	(PC00181)	3	1.10%	1.00%
19	transcription factor	(PC00218)	7	2.70%	2.40%
20	chaperone	(PC00072)	5	1.90%	1.70%
21	cell junction protein	(PC00070)	2	0.80%	0.70%
22	surfactant	(PC00212)	2	0.80%	0.70%
23	structural protein	(PC00211)	2	0.80%	0.70%
24	kinase	(PC00137)	8	3.00%	2.80%
25	storage protein	(PC00210)	1	0.40%	0.30%
26	receptor	(PC00197)	27	10.30%	9.30%
27	isomerase	(PC00135)	1	0.40%	0.30%

B. Overrepresentation test complete

**GO molecular function complete**	**Homo sapiens - REFLIST (20814)**	**Client Text Box Input (260)**	**Client Text Box Input (expected)**	**Client Text Box Input (over/under)**	**Client Text Box Input (fold Enrichment)**	**Client Text Box Input (P-value)**
oxidoreductase activity (GO:0016491)	742	25	9.27	+	2.7	2.11E-02
protein complex binding (GO:0032403)	898	30	11.22	+	2.67	2.98E-03
identical protein binding (GO:0042802)	1173	34	14.65	+	2.32	1.24E-02
protein binding (GO:0005515)	10420	179	130.16	+	1.38	1.59E-06
binding (GO:0005488)	13955	209	174.32	+	1.2	3.10E-03
molecular_function (GO:0003674)	16648	235	207.96	+	1.13	1.06E-02
Unclassified (UNCLASSIFIED)	4166	27	52.04	-	0.52	0.00E+00
**GO biological process complete**	**Homo sapiens - REFLIST (20814)**	**Client Text Box Input**	**Client Text Box Input (expected)**	**Client Text Box Input (over/under)**	**Client Text Box Input (fold Enrichment)**	**Client Text Box Input (P-value)**

		**(260)**				
glial cell development (GO:0021782)	75	8	0.94	+	> 5	4.59E-02
glial cell differentiation (GO:0010001)	136	11	1.7	+	> 5	1.21E-02
regeneration (GO:0031099)	148	11	1.85	+	> 5	2.69E-02
response to wounding (GO:0009611)	738	30	9.22	+	3.25	1.52E-04
vesicle-mediated transport (GO:0016192)	1150	36	14.37	+	2.51	3.44E-03
oxidation-reduction process (GO:0055114)	1034	32	12.92	+	2.48	2.03E-02
regulation of cellular component organization (GO:0051128)	2160	56	26.98	+	2.08	8.36E-04
establishment of localization (GO:0051234)	3851	85	48.11	+	1.77	2.52E-04
regulation of biological quality (GO:0065008)	3134	69	39.15	+	1.76	9.59E-03
transport (GO:0006810)	3742	82	46.74	+	1.75	6.94E-04
single-organism localization (GO:1902578)	3296	72	41.17	+	1.75	6.84E-03
response to stress (GO:0006950)	3648	79	45.57	+	1.73	2.21E-03
localization (GO:0051179)	4683	99	58.5	+	1.69	8.61E-05
single-organism metabolic process (GO:0044710)	4494	92	56.14	+	1.64	1.89E-03
single-organism developmental process (GO:0044767)	5209	98	65.07	+	1.51	3.33E-02
response to stimulus (GO:0050896)	7621	138	95.2	+	1.45	3.69E-04
single-organism process (GO:0044699)	12755	210	159.33	+	1.32	7.97E-08
single-organism cellular process (GO:0044763)	11415	186	142.59	+	1.3	1.88E-04
cellular process (GO:0009987)	14147	209	176.72	+	1.18	4.19E-02

biological_process (GO:0008150)	16542	240	206.64	+	1.16	8.78E-05
RNA metabolic process (GO:0016070)	3373	18	42.13	-	0.43	4.85E-02
Unclassified (UNCLASSIFIED)	4272	22	53.36	-	0.41	0.00E+00
**PANTHER Protein Class**	**Homo sapiens - REFLIST (20814)**	**Client Text Box Input (260)**	**Client Text Box Input (expected)**	**Client Text Box Input (over/under)**	**Client Text Box Input (fold Enrichment)**	**Client Text Box Input (P-value)**
actin family cytoskeletal protein (PC00041)	395	17	4.93	+	3.45	2.60E-03
oxidoreductase (PC00176)	609	20	7.61	+	2.63	2.01E-02
cytoskeletal protein (PC00085)	806	25	10.07	+	2.48	6.82E-03
Unclassified (UNCLASSIFIED)	9675	85	120.86	-	0.7	0.00E+00

C. Overrepresentation test GO-slim

**PANTHER GO-Slim Molecular Function**	**Homo sapiens - REFLIST (20814)**	**Client Text Box Input (260)**	**Client Text Box Input (expected)**	**Client Text Box Input (over/under)**	**Client Text Box Input (fold Enrichment)**	**Client Text Box Input (P-value)**
structural constituent of cytoskeleton (GO:0005200)	663	22	8.28	+	2.66	6.44E-03
oxidoreductase activity (GO:0016491)	626	20	7.82	+	2.56	2.41E-02
catalytic activity (GO:0003824)	5209	91	65.07	+	1.4	3.64E-02
Unclassified (UNCLASSIFIED)	10020	95	125.17	-	0.76	0.00E+00
DNA binding (GO:0003677)	1878	8	23.46	-	0.34	2.21E-02
nucleic acid binding transcription factor activity (GO:0001071)	1646	6	20.56	-	0.29	1.84E-02
**PANTHER GO-Slim Biological Process**	**Homo sapiens - REFLIST (20814)**	**Client Text Box Input (260)**	**Client Text Box Input (expected)**	**Client Text Box Input (over/under)**	**Client Text Box Input (fold Enrichment)**	**Client Text Box Input (P-value)**
cellular component organization or biogenesis (GO:0071840)	1316	33	16.44	+	2.01	2.64E-02
Unclassified (UNCLASSIFIED)	8629	77	107.79	-	0.71	0.00E+00

regulation of transcription from RNA polymerase II promoter (GO:0006357)	1319	4	16.48	-	0.24	4.45E-02
regulation of nucleobase-containing compound metabolic process (GO:0019219)	1700	5	21.24	-	0.24	3.54E-03
