# Supplementary material for: When the brain goes diving: transcriptome analysis reveals a reduced aerobic energy metabolism and increased stress proteins in the seal brain
Source: BMC Genomics. 2016 Aug 9;17:583. doi: 10.1186/s12864-016-2892-y (PMC4979143; doi:10.1186/s12864-016-2892-y)
Supplement: Additional file 5: Table S4. — Genes overrepresented in the ferret brain. Ontology analysis of genes that are at least twofold higher expressed in the visual cortex of the ferret compared to the hooded seal visual cortex. A. The GO terms for the domains “molecular function” and “biological process”, and the “protein class” are given. B and C. PANTHER Overrepresentation Test of the domains “molecular function”, “biological process”, and “protein class” using the complete (B) and PANTHER GO-slim terms. (PDF 74 kb) [file 12864_2016_2892_MOESM5_ESM.pdf]

**Additional File 5: Table S4. Genes overrepresented in the ferret brain.** Ontology analysis of genes that are at least twofold higher expressed in the visual cortex of the ferret compared to the hooded seal visual cortex. A. The GO terms for the domains "molecular function" and "biological process", and the "protein class" are given. B and C. PANTHER Overrepresentation Test of the domains "molecular function", "biological process", and "protein class" using the complete (B) and PANTHER GO-slim terms.

| **A** | Category name | Accession | number of genes | Percent of gene hit against total | Function hits |
|---|---|---|---|---|---|
| **Molecular function** | | | | | |
| 1 | protein binding transcription factor | (GO:0000988) | 9 | 0.80% | 0.80% |
| 2 | nucleic acid binding transcription factor | (GO:0001071) | 44 | 3.70% | 4.10% |
| 3 | catalytic activity | (GO:0003824) | 374 | 31.60% | 34.80% |
| 4 | receptor | (GO:0004872) | 41 | 3.50% | 3.80% |
| 5 | structural molecule | (GO:0005198) | 103 | 8.70% | 9.60% |
| 6 | transporter | (GO:0005215) | 68 | 5.70% | 6.30% |
| 7 | binding | (GO:0005488) | 351 | 29.70% | 32.60% |
| 8 | antioxidant | (GO:0016209) | 4 | 0.30% | 0.40% |
| 9 | channel regulator | (GO:0016247) | 1 | 0.10% | 0.10% |
| 10 | enzyme regulator | (GO:0030234) | 63 | 5.30% | 5.90% |
| 11 | translation regulator | (GO:0045182) | 18 | 1.50% | 1.70% |
| | | | | | |
| **Biological process** | | | | | |
| 1 | cellular component organization or biogenesis | (GO:0071840) | 107 | 9.00% | 6.00% |
| 2 | cellular process | (GO:0009987) | 384 | 32.50% | 21.40% |
| 3 | localization | (GO:0051179) | 188 | 15.90% | 10.50% |
| 4 | apoptotic process | (GO:0006915) | 23 | 1.90% | 1.30% |
| 5 | reproduction | (GO:0000003) | 16 | 1.40% | 0.90% |
| 6 | biological regulation | (GO:0065007) | 191 | 16.10% | 10.60% |
| 7 | response to stimulus | (GO:0050896) | 68 | 5.70% | 3.80% |
| 8 | developmental process | (GO:0032502) | 95 | 8.00% | 5.30% |
| 9 | multicellular organismal process | (GO:0032501) | 83 | 7.00% | 4.60% |
| 10 | locomotion | (GO:0040011) | 1 | 0.10% | 0.10% |
| 11 | biological adhesion | (GO:0022610) | 21 | 1.80% | 1.20% |
| 12 | metabolic process | (GO:0008152) | 575 | 48.60% | 32.00% |
| 13 | growth | (GO:0040007) | 2 | 0.20% | 0.10% |
| 14 | immune system process | (GO:0002376) | 44 | 3.70% | 2.40% |
| | | | | | |
| **Protein class** | | | | | |
| 1 | extracellular matrix protein | (PC00102) | 7 | 0.60% | 0.70% |
| 2 | protease | (PC00190) | 16 | 1.40% | 1.50% |
| 3 | cytoskeletal protein | (PC00085) | 41 | 3.50% | 3.90% |

| | | | | | |
|---|---|---|---|---|---|
| 4 | transporter | (PC00227) | 66 | 5.60% | 6.30% |
| 5 | transmembrane receptor regulatory/adaptor protein | (PC00226) | 5 | 0.40% | 0.50% |
| 6 | transferase | (PC00220) | 83 | 7.00% | 7.90% |
| 7 | oxidoreductase | (PC00176) | 62 | 5.20% | 5.90% |
| 8 | lyase | (PC00144) | 10 | 0.80% | 1.00% |
| 9 | cell adhesion molecule | (PC00069) | 11 | 0.90% | 1.00% |
| 10 | ligase | (PC00142) | 26 | 2.20% | 2.50% |
| 11 | nucleic acid binding | (PC00171) | 181 | 15.30% | 17.30% |
| 12 | signaling molecule | (PC00207) | 44 | 3.70% | 4.20% |
| 13 | enzyme modulator | (PC00095) | 86 | 7.30% | 8.20% |
| 14 | calcium-binding protein | (PC00060) | 37 | 3.10% | 3.50% |
| 15 | defense/immunity protein | (PC00090) | 8 | 0.70% | 0.80% |
| 16 | hydrolase | (PC00121) | 75 | 6.30% | 7.10% |
| 17 | transfer/carrier protein | (PC00219) | 33 | 2.80% | 3.10% |
| 18 | membrane traffic protein | (PC00150) | 58 | 4.90% | 5.50% |
| 19 | phosphatase | (PC00181) | 17 | 1.40% | 1.60% |
| 20 | transcription factor | (PC00218) | 47 | 4.00% | 4.50% |
| 21 | chaperone | (PC00072) | 27 | 2.30% | 2.60% |
| 22 | cell junction protein | (PC00070) | 4 | 0.30% | 0.40% |
| 23 | surfactant | (PC00212) | 2 | 0.20% | 0.20% |
| 24 | structural protein | (PC00211) | 2 | 0.20% | 0.20% |
| 25 | kinase | (PC00137) | 41 | 3.50% | 3.90% |
| 26 | storage protein | (PC00210) | 1 | 0.10% | 0.10% |
| 27 | receptor | (PC00197) | 45 | 3.80% | 4.30% |
| 28 | isomerase | (PC00135) | 14 | 1.20% | 1.30% |

**B. Overrepresentation test complete**

| **GO molecular function complete** | **Homo sapiens - REFLIST (20814)** | **Client Text Box Input (1175)** | **Client Text Box Input (expected)** | **Client Text Box Input (over/under)** | **Client Text Box Input (fold Enrichment)** | **Client Text Box Input (P-value)** |
|---|---|---|---|---|---|---|
| structural constituent of ribosome (GO:0003735) | 163 | 70 | 9.2 | + | > 5 | 1.43E-34 |
| proton-transporting ATPase activity, rotational mechanism (GO:0046961) | 22 | 9 | 1.24 | + | > 5 | 1.53E-02 |
| ATPase activity, coupled to transmembrane movement of ions, rotational mechanism (GO:0044769) | 25 | 10 | 1.41 | + | > 5 | 5.74E-03 |
| NADH dehydrogenase (quinone) activity (GO:0050136) | 47 | 18 | 2.65 | + | > 5 | 1.21E-06 |
| NADH dehydrogenase (ubiquinone) activity (GO:0008137) | 47 | 18 | 2.65 | + | > 5 | 1.21E-06 |
| NADH dehydrogenase activity (GO:0003954) | 48 | 18 | 2.71 | + | > 5 | 1.67E-06 |
| hydrogen-exporting ATPase activity (GO:0036442) | 27 | 10 | 1.52 | + | > 5 | 1.12E-02 |
| SNAP receptor activity | 39 | 13 | 2.2 | + | > 5 | 1.40E-03 |

| | | | | | | |
|---|---|---|---|---|---|---|
| (GO:0005484) | | | | | | |
| oxidoreductase activity, acting on NAD(P)H, quinone or similar compound as acceptor (GO:0016655) | 61 | 18 | 3.44 | + | > 5 | 6.33E-05 |
| hydrogen ion transmembrane transporter activity (GO:0015078) | 111 | 29 | 6.27 | + | 4.63 | 6.94E-08 |
| cation-transporting ATPase activity (GO:0019829) | 54 | 14 | 3.05 | + | 4.59 | 9.50E-03 |
| ATPase activity, coupled to transmembrane movement of ions (GO:0042625) | 55 | 14 | 3.1 | + | 4.51 | 1.17E-02 |
| SNARE binding (GO:0000149) | 119 | 28 | 6.72 | + | 4.17 | 1.52E-06 |
| syntaxin binding (GO:0019905) | 84 | 19 | 4.74 | + | 4.01 | 1.47E-03 |
| ion channel binding (GO:0044325) | 98 | 20 | 5.53 | + | 3.62 | 3.56E-03 |
| oxidoreductase activity, acting on NAD(P)H (GO:0016651) | 105 | 21 | 5.93 | + | 3.54 | 2.69E-03 |
| translation factor activity, RNA binding (GO:0008135) | 107 | 19 | 6.04 | + | 3.15 | 4.40E-02 |
| structural molecule activity (GO:0005198) | 639 | 90 | 36.07 | + | 2.49 | 2.26E-11 |
| poly(A) RNA binding (GO:0044822) | 1152 | 153 | 65.03 | + | 2.35 | 8.71E-19 |
| RNA binding (GO:0003723) | 1559 | 194 | 88.01 | + | 2.2 | 1.68E-21 |
| monovalent inorganic cation transmembrane transporter activity (GO:0015077) | 373 | 43 | 21.06 | + | 2.04 | 3.58E-02 |
| protein domain specific binding (GO:0019904) | 591 | 63 | 33.36 | + | 1.89 | 5.16E-03 |
| oxidoreductase activity (GO:0016491) | 742 | 79 | 41.89 | + | 1.89 | 2.75E-04 |
| hydrolase activity, acting on acid anhydrides, in phosphorus-containing anhydrides (GO:0016818) | 815 | 80 | 46.01 | + | 1.74 | 5.14E-03 |
| hydrolase activity, acting on acid anhydrides (GO:0016817) | 817 | 80 | 46.12 | + | 1.73 | 5.62E-03 |
| pyrophosphatase activity (GO:0016462) | 812 | 79 | 45.84 | + | 1.72 | 8.19E-03 |
| identical protein binding (GO:0042802) | 1173 | 108 | 66.22 | + | 1.63 | 1.67E-03 |
| enzyme binding (GO:0019899) | 1604 | 143 | 90.55 | + | 1.58 | 1.48E-04 |
| catalytic activity (GO:0003824) | 5667 | 441 | 319.92 | + | 1.38 | 2.42E-11 |
| nucleoside phosphate binding (GO:1901265) | 2326 | 180 | 131.31 | + | 1.37 | 2.43E-02 |
| nucleotide binding (GO:0000166) | 2325 | 179 | 131.25 | + | 1.36 | 3.45E-02 |
| small molecule binding (GO:0036094) | 2593 | 198 | 146.38 | + | 1.35 | 1.83E-02 |
| protein binding (GO:0005515) | 10420 | 779 | 588.23 | + | 1.32 | 4.83E-26 |
| heterocyclic compound binding (GO:1901363) | 5826 | 401 | 328.89 | + | 1.22 | 6.09E-03 |

| | | | | | | |
|---|---|---|---|---|---|---|
| organic cyclic compound binding (GO:0097159) | 5899 | 406 | 333.01 | + | 1.22 | 5.04E-03 |
| binding (GO:0005488) | 13955 | 950 | 787.79 | + | 1.21 | 8.77E-23 |
| molecular_function (GO:0003674) | 16648 | 1072 | 939.82 | + | 1.14 | 7.55E-23 |
| DNA binding (GO:0003677) | 2421 | 73 | 136.67 | - | 0.53 | 5.86E-07 |
| molecular transducer activity (GO:0060089) | 1848 | 48 | 104.32 | - | 0.46 | 3.09E-07 |
| Unclassified (UNCLASSIFIED) | 4166 | 106 | 235.18 | - | 0.45 | 0.00E+00 |
| nucleic acid binding transcription factor activity (GO:0001071) | 1166 | 29 | 65.82 | - | 0.44 | 3.67E-04 |
| transcription factor activity, sequence-specific DNA binding (GO:0003700) | 1166 | 29 | 65.82 | - | 0.44 | 3.67E-04 |
| signal transducer activity (GO:0004871) | 1644 | 40 | 92.81 | - | 0.43 | 3.60E-07 |
| receptor activity (GO:0004872) | 1567 | 35 | 88.46 | - | 0.4 | 5.47E-08 |
| signaling receptor activity (GO:0038023) | 1360 | 27 | 76.78 | - | 0.35 | 4.04E-08 |
| transmembrane signaling receptor activity (GO:0004888) | 1259 | 24 | 71.07 | - | 0.34 | 8.14E-08 |
| G-protein coupled receptor activity (GO:0004930) | 868 | 12 | 49 | - | 0.24 | 3.68E-07 |
| response to stimulus (GO:0050896) | 2170 | 68 | 122.5 | 0.56 | - | 2.95E-06 |
| transcription, DNA-dependent (GO:0006351) | 1941 | 60 | 109.57 | 0.55 | - | 1.12E-05 |
| regulation of transcription from RNA polymerase II promoter (GO:0006357) | 1319 | 40 | 74.46 | 0.54 | - | 1.13E-03 |
| regulation of nucleobase-containing compound metabolic process (GO:0019219) | 1700 | 50 | 95.97 | 0.52 | - | 1.48E-05 |
| mesoderm development (GO:0007498) | 671 | 17 | 37.88 | 0.45 | - | 2.16E-02 |
| ectoderm development (GO:0007398) | 663 | 15 | 37.43 | 0.4 | - | 4.83E-03 |
| sensory perception (GO:0007600) | 455 | 9 | 25.69 | 0.35 | - | 2.74E-02 |
| cell-cell adhesion (GO:0016337) | 391 | 7 | 22.07 | 0.32 | - | 3.75E-02 |
| skeletal system development (GO:0001501) | 232 | 1 | 13.1 | < 0.2 | - | 6.00E-03 |
| **GO biological process complete** | **Homo sapiens - REFLIST (20814)** | **Client Text Box Input (1175)** | **Client Text Box Input (expected)** | **Client Text Box Input (over/under)** | **Client Text Box Input (fold Enrichment)** | **Client Text Box Input (P-value)** |
| mitochondrial ATP synthesis coupled proton transport (GO:0042776) | 19 | 9 | 1.07 | + | > 5 | 1.50E-02 |
| translational termination (GO:0006415) | 171 | 73 | 9.65 | + | > 5 | 1.73E-35 |
| SRP-dependent cotranslational protein | 109 | 46 | 6.15 | + | > 5 | 3.51E-21 |

| | | | | | | |
|---|---|---|---|---|---|---|
| targeting to membrane (GO:0006614) | | | | | | |
| cytoplasmic translation (GO:0002181) | 31 | 13 | 1.75 | + | > 5 | 3.40E-04 |
| viral transcription (GO:0019083) | 111 | 46 | 6.27 | + | > 5 | 7.29E-21 |
| cotranslational protein targeting to membrane (GO:0006613) | 112 | 46 | 6.32 | + | > 5 | 1.04E-20 |
| ribosomal large subunit biogenesis (GO:0042273) | 39 | 16 | 2.2 | + | > 5 | 1.33E-05 |
| translational elongation (GO:0006414) | 200 | 82 | 11.29 | + | > 5 | 5.69E-39 |
| protein targeting to ER (GO:0045047) | 113 | 46 | 6.38 | + | > 5 | 1.49E-20 |
| establishment of protein localization to endoplasmic reticulum (GO:0072599) | 117 | 47 | 6.6 | + | > 5 | 8.07E-21 |
| mitochondrial ATP synthesis coupled electron transport (GO:0042775) | 68 | 27 | 3.84 | + | > 5 | 8.45E-11 |
| ATP synthesis coupled electron transport (GO:0042773) | 68 | 27 | 3.84 | + | > 5 | 8.45E-11 |
| mitochondrial electron transport, NADH to ubiquinone (GO:0006120) | 46 | 18 | 2.6 | + | > 5 | 2.74E-06 |
| cellular protein complex disassembly (GO:0043624) | 200 | 78 | 11.29 | + | > 5 | 1.89E-35 |
| viral gene expression (GO:0019080) | 121 | 46 | 6.83 | + | > 5 | 2.26E-19 |
| glutamate secretion (GO:0014047) | 29 | 11 | 1.64 | + | > 5 | 9.59E-03 |
| nuclear-transcribed mRNA catabolic process, nonsense-mediated decay (GO:0000184) | 119 | 45 | 6.72 | + | > 5 | 8.31E-19 |
| oxidative phosphorylation (GO:0006119) | 77 | 29 | 4.35 | + | > 5 | 3.32E-11 |
| multi-organism metabolic process (GO:0044033) | 127 | 47 | 7.17 | + | > 5 | 2.24E-19 |
| respiratory electron transport chain (GO:0022904) | 135 | 49 | 7.62 | + | > 5 | 5.91E-20 |
| electron transport chain (GO:0022900) | 137 | 49 | 7.73 | + | > 5 | 1.09E-19 |
| protein complex disassembly (GO:0043241) | 220 | 78 | 12.42 | + | > 5 | 1.12E-32 |
| translational initiation (GO:0006413) | 232 | 82 | 13.1 | + | > 5 | 2.06E-34 |
| protein localization to endoplasmic reticulum (GO:0070972) | 136 | 48 | 7.68 | + | > 5 | 5.34E-19 |
| mitochondrial translational elongation (GO:0070125) | 84 | 29 | 4.74 | + | > 5 | 2.86E-10 |
| mitochondrial translational initiation (GO:0070124) | 84 | 29 | 4.74 | + | > 5 | 2.86E-10 |
| macromolecular complex disassembly (GO:0032984) | 229 | 79 | 12.93 | + | > 5 | 2.45E-32 |
| mitochondrial translational termination (GO:0070126) | 86 | 29 | 4.85 | + | > 5 | 5.08E-10 |

| | | | | | | |
|---|---|---|---|---|---|---|
| mitochondrial translation (GO:0032543) | 107 | 35 | 6.04 | + | > 5 | 3.29E-12 |
| cellular respiration (GO:0045333) | 185 | 59 | 10.44 | + | > 5 | 9.33E-22 |
| nuclear-transcribed mRNA catabolic process (GO:0000956) | 181 | 56 | 10.22 | + | > 5 | 6.64E-20 |
| spliceosomal snRNP assembly (GO:0000387) | 39 | 12 | 2.2 | + | > 5 | 2.69E-02 |
| mRNA catabolic process (GO:0006402) | 193 | 57 | 10.9 | + | > 5 | 2.34E-19 |
| mitochondrial transmembrane transport (GO:1990542) | 51 | 15 | 2.88 | + | > 5 | 2.97E-03 |
| ribosome assembly (GO:0042255) | 51 | 15 | 2.88 | + | > 5 | 2.97E-03 |
| RNA catabolic process (GO:0006401) | 222 | 61 | 12.53 | + | 4.87 | 2.41E-19 |
| protein targeting to membrane (GO:0006612) | 186 | 51 | 10.5 | + | 4.86 | 1.03E-15 |
| translation (GO:0006412) | 397 | 108 | 22.41 | + | 4.82 | 3.83E-36 |
| ATP metabolic process (GO:0046034) | 178 | 48 | 10.05 | + | 4.78 | 2.33E-14 |
| purine ribonucleoside triphosphate metabolic process (GO:0009205) | 193 | 52 | 10.9 | + | 4.77 | 9.44E-16 |
| purine nucleoside triphosphate metabolic process (GO:0009144) | 201 | 53 | 11.35 | + | 4.67 | 1.05E-15 |
| aerobic respiration (GO:0009060) | 57 | 15 | 3.22 | + | 4.66 | 1.15E-02 |
| ribonucleoside triphosphate metabolic process (GO:0009199) | 199 | 52 | 11.23 | + | 4.63 | 3.37E-15 |
| peptide biosynthetic process (GO:0043043) | 420 | 109 | 23.71 | + | 4.6 | 1.03E-34 |
| hydrogen ion transmembrane transport (GO:1902600) | 116 | 30 | 6.55 | + | 4.58 | 1.29E-07 |
| purine ribonucleoside monophosphate metabolic process (GO:0009167) | 202 | 49 | 11.4 | + | 4.3 | 6.30E-13 |
| purine nucleoside monophosphate metabolic process (GO:0009126) | 203 | 49 | 11.46 | + | 4.28 | 7.60E-13 |
| nucleoside triphosphate metabolic process (GO:0009141) | 220 | 53 | 12.42 | + | 4.27 | 4.58E-14 |
| dicarboxylic acid transport (GO:0006835) | 63 | 15 | 3.56 | + | 4.22 | 3.80E-02 |
| amide biosynthetic process (GO:0043604) | 480 | 114 | 27.1 | + | 4.21 | 5.41E-33 |
| ribonucleoside monophosphate metabolic process (GO:0009161) | 215 | 51 | 12.14 | + | 4.2 | 3.58E-13 |
| viral life cycle (GO:0019058) | 241 | 57 | 13.61 | + | 4.19 | 5.75E-15 |
| establishment of protein localization to membrane (GO:0090150) | 273 | 64 | 15.41 | + | 4.15 | 6.19E-17 |
| peptide metabolic process (GO:0006518) | 517 | 117 | 29.19 | + | 4.01 | 4.75E-32 |
| nucleoside monophosphate | 226 | 51 | 12.76 | + | 4 | 2.54E-12 |

| | | | | | | |
|---|---|---|---|---|---|---|
| metabolic process (GO:0009123) | | | | | | |
| energy derivation by oxidation of organic compounds (GO:0015980) | 342 | 76 | 19.31 | + | 3.94 | 2.90E-19 |
| neurotransmitter secretion (GO:0007269) | 122 | 27 | 6.89 | + | 3.92 | 3.45E-05 |
| regulation of synaptic plasticity (GO:0048167) | 133 | 29 | 7.51 | + | 3.86 | 1.30E-05 |
| establishment of protein localization to organelle (GO:0072594) | 348 | 74 | 19.65 | + | 3.77 | 1.30E-17 |
| neurotransmitter transport (GO:0006836) | 160 | 34 | 9.03 | + | 3.76 | 1.02E-06 |
| mitochondrial transport (GO:0006839) | 153 | 32 | 8.64 | + | 3.7 | 5.18E-06 |
| ribonucleoprotein complex assembly (GO:0022618) | 182 | 38 | 10.27 | + | 3.7 | 1.40E-07 |
| ribonucleoprotein complex subunit organization (GO:0071826) | 192 | 40 | 10.84 | + | 3.69 | 4.44E-08 |
| proton transport (GO:0015992) | 153 | 31 | 8.64 | + | 3.59 | 1.98E-05 |
| generation of precursor metabolites and energy (GO:0006091) | 411 | 83 | 23.2 | + | 3.58 | 1.04E-18 |
| ribosome biogenesis (GO:0042254) | 218 | 44 | 12.31 | + | 3.58 | 1.10E-08 |
| protein localization to membrane (GO:0072657) | 388 | 78 | 21.9 | + | 3.56 | 2.69E-17 |
| nucleobase-containing compound catabolic process (GO:0034655) | 344 | 69 | 19.42 | + | 3.55 | 6.16E-15 |
| hydrogen transport (GO:0006818) | 155 | 31 | 8.75 | + | 3.54 | 2.66E-05 |
| synaptic vesicle localization (GO:0097479) | 110 | 22 | 6.21 | + | 3.54 | 4.79E-03 |
| synaptic vesicle transport (GO:0048489) | 108 | 21 | 6.1 | + | 3.44 | 1.32E-02 |
| establishment of synaptic vesicle localization (GO:0097480) | 108 | 21 | 6.1 | + | 3.44 | 1.32E-02 |
| ribonucleoprotein complex biogenesis (GO:0022613) | 346 | 67 | 19.53 | + | 3.43 | 1.12E-13 |
| ribonucleotide metabolic process (GO:0009259) | 324 | 62 | 18.29 | + | 3.39 | 3.14E-12 |
| purine ribonucleoside metabolic process (GO:0046128) | 277 | 53 | 15.64 | + | 3.39 | 4.43E-10 |
| purine ribonucleotide metabolic process (GO:0009150) | 309 | 59 | 17.44 | + | 3.38 | 1.80E-11 |
| rRNA processing (GO:0006364) | 147 | 28 | 8.3 | + | 3.37 | 4.06E-04 |
| mitochondrion organization (GO:0007005) | 410 | 78 | 23.15 | + | 3.37 | 6.21E-16 |
| ribose phosphate metabolic process (GO:0019693) | 337 | 64 | 19.02 | + | 3.36 | 1.48E-12 |
| purine nucleoside metabolic process (GO:0042278) | 280 | 53 | 15.81 | + | 3.35 | 6.69E-10 |

| | | | | | | |
|---|---|---|---|---|---|---|
| cellular amide metabolic process (GO:0043603) | 666 | 125 | 37.6 | + | 3.32 | 7.06E-27 |
| regulation of neurotransmitter levels (GO:0001505) | 155 | 29 | 8.75 | + | 3.31 | 3.43E-04 |
| heterocycle catabolic process (GO:0046700) | 386 | 72 | 21.79 | + | 3.3 | 4.71E-14 |
| protein targeting (GO:0006605) | 423 | 78 | 23.88 | + | 3.27 | 3.56E-15 |
| mRNA metabolic process (GO:0016071) | 564 | 104 | 31.84 | + | 3.27 | 3.25E-21 |
| aromatic compound catabolic process (GO:0019439) | 396 | 73 | 22.36 | + | 3.27 | 5.09E-14 |
| purine nucleotide metabolic process (GO:0006163) | 331 | 61 | 18.69 | + | 3.26 | 2.83E-11 |
| rRNA metabolic process (GO:0016072) | 152 | 28 | 8.58 | + | 3.26 | 7.95E-04 |
| cellular nitrogen compound catabolic process (GO:0044270) | 386 | 71 | 21.79 | + | 3.26 | 1.63E-13 |
| organic cyclic compound catabolic process (GO:1901361) | 427 | 78 | 24.11 | + | 3.24 | 6.01E-15 |
| cellular component disassembly (GO:0022411) | 493 | 89 | 27.83 | + | 3.2 | 3.93E-17 |
| ribonucleoside metabolic process (GO:0009119) | 301 | 54 | 16.99 | + | 3.18 | 3.05E-09 |
| regulation of synapse structure or activity (GO:0050803) | 218 | 37 | 12.31 | + | 3.01 | 6.28E-05 |
| nucleoside metabolic process (GO:0009116) | 326 | 55 | 18.4 | + | 2.99 | 1.93E-08 |
| mRNA splicing, via spliceosome (GO:0000398) | 226 | 38 | 12.76 | + | 2.98 | 5.06E-05 |
| RNA splicing, via transesterification reactions with bulged adenosine as nucleophile (GO:0000377) | 226 | 38 | 12.76 | + | 2.98 | 5.06E-05 |
| establishment of vesicle localization (GO:0051650) | 186 | 31 | 10.5 | + | 2.95 | 1.46E-03 |
| RNA splicing, via transesterification reactions (GO:0000375) | 229 | 38 | 12.93 | + | 2.94 | 7.11E-05 |
| nucleotide metabolic process (GO:0009117) | 484 | 80 | 27.32 | + | 2.93 | 5.51E-13 |
| vesicle localization (GO:0051648) | 194 | 32 | 10.95 | + | 2.92 | 1.17E-03 |
| purine-containing compound metabolic process (GO:0072521) | 378 | 62 | 21.34 | + | 2.91 | 2.52E-09 |
| single-organism cellular localization (GO:1902580) | 845 | 138 | 47.7 | + | 2.89 | 3.54E-24 |
| nucleoside phosphate metabolic process (GO:0006753) | 490 | 80 | 27.66 | + | 2.89 | 1.08E-12 |
| protein localization to organelle (GO:0033365) | 528 | 86 | 29.81 | + | 2.89 | 8.52E-14 |
| glycosyl compound metabolic process (GO:1901657) | 344 | 56 | 19.42 | + | 2.88 | 4.73E-08 |
| Golgi vesicle transport (GO:0048193) | 246 | 39 | 13.89 | + | 2.81 | 1.51E-04 |

| | | | | | | |
|---|---|---|---|---|---|---|
| organonitrogen compound biosynthetic process (GO:1901566) | 1007 | 159 | 56.85 | + | 2.8 | 6.93E-27 |
| positive regulation of protein ubiquitination (GO:0031398) | 165 | 26 | 9.31 | + | 2.79 | 3.69E-02 |
| signal release (GO:0023061) | 189 | 29 | 10.67 | + | 2.72 | 1.79E-02 |
| single-organism membrane organization (GO:0044802) | 715 | 109 | 40.36 | + | 2.7 | 3.45E-16 |
| vesicle organization (GO:0016050) | 214 | 32 | 12.08 | + | 2.65 | 9.38E-03 |
| membrane organization (GO:0061024) | 877 | 131 | 49.51 | + | 2.65 | 2.30E-19 |
| cellular macromolecule catabolic process (GO:0044265) | 764 | 114 | 43.13 | + | 2.64 | 2.21E-16 |
| intracellular protein transport (GO:0006886) | 727 | 108 | 41.04 | + | 2.63 | 3.21E-15 |
| nucleobase-containing small molecule metabolic process (GO:0055086) | 547 | 81 | 30.88 | + | 2.62 | 1.27E-10 |
| modulation of synaptic transmission (GO:0050804) | 266 | 39 | 15.02 | + | 2.6 | 1.10E-03 |
| protein polyubiquitination (GO:0000209) | 198 | 29 | 11.18 | + | 2.59 | 4.28E-02 |
| cytoplasmic transport (GO:0016482) | 713 | 104 | 40.25 | + | 2.58 | 5.18E-14 |
| viral process (GO:0016032) | 674 | 98 | 38.05 | + | 2.58 | 6.03E-13 |
| multi-organism cellular process (GO:0044764) | 678 | 98 | 38.27 | + | 2.56 | 8.73E-13 |
| interspecies interaction between organisms (GO:0044419) | 749 | 108 | 42.28 | + | 2.55 | 2.53E-14 |
| symbiosis, encompassing mutualism through parasitism (GO:0044403) | 749 | 108 | 42.28 | + | 2.55 | 2.53E-14 |
| single-organism intracellular transport (GO:1902582) | 1168 | 165 | 65.94 | + | 2.5 | 8.35E-23 |
| RNA splicing (GO:0008380) | 338 | 46 | 19.08 | + | 2.41 | 7.21E-04 |
| regulation of cellular amide metabolic process (GO:0034248) | 311 | 42 | 17.56 | + | 2.39 | 3.14E-03 |
| regulation of translation (GO:0006417) | 289 | 39 | 16.31 | + | 2.39 | 8.20E-03 |
| exocytosis (GO:0006887) | 297 | 40 | 16.77 | + | 2.39 | 6.29E-03 |
| neurotrophin signaling pathway (GO:0038179) | 397 | 53 | 22.41 | + | 2.36 | 1.43E-04 |
| neurotrophin TRK receptor signaling pathway (GO:0048011) | 394 | 52 | 22.24 | + | 2.34 | 2.78E-04 |
| intracellular transport (GO:0046907) | 1353 | 178 | 76.38 | + | 2.33 | 1.72E-21 |
| macromolecule catabolic process (GO:0009057) | 899 | 118 | 50.75 | + | 2.33 | 5.94E-13 |
| organonitrogen compound metabolic process (GO:1901564) | 1727 | 226 | 97.49 | + | 2.32 | 2.44E-28 |
| protein transport (GO:0015031) | 1224 | 160 | 69.1 | + | 2.32 | 1.26E-18 |
| establishment of protein localization (GO:0045184) | 1307 | 170 | 73.78 | + | 2.3 | 8.09E-20 |

| | | | | | | |
|---|---|---|---|---|---|---|
| cellular macromolecular complex assembly (GO:0034622) | 608 | 79 | 34.32 | + | 2.3 | 1.59E-07 |
| organophosphate metabolic process (GO:0019637) | 834 | 108 | 47.08 | + | 2.29 | 3.22E-11 |
| establishment of organelle localization (GO:0051656) | 294 | 38 | 16.6 | + | 2.29 | 2.99E-02 |
| mRNA processing (GO:0006397) | 396 | 51 | 22.36 | + | 2.28 | 7.86E-04 |
| cellular protein localization (GO:0034613) | 1138 | 145 | 64.24 | + | 2.26 | 1.47E-15 |
| cellular macromolecule localization (GO:0070727) | 1146 | 145 | 64.69 | + | 2.24 | 2.68E-15 |
| organelle localization (GO:0051640) | 368 | 46 | 20.77 | + | 2.21 | 7.30E-03 |
| ncRNA metabolic process (GO:0034660) | 409 | 51 | 23.09 | + | 2.21 | 2.04E-03 |
| establishment of localization in cell (GO:0051649) | 1812 | 219 | 102.29 | + | 2.14 | 1.04E-22 |
| oxidation-reduction process (GO:0055114) | 1034 | 123 | 58.37 | + | 2.11 | 1.40E-10 |
| protein localization (GO:0008104) | 1689 | 200 | 95.35 | + | 2.1 | 2.83E-19 |
| synaptic transmission (GO:0007268) | 560 | 66 | 31.61 | + | 2.09 | 2.91E-04 |
| secretion by cell (GO:0032940) | 484 | 57 | 27.32 | + | 2.09 | 2.53E-03 |
| protein complex subunit organization (GO:0071822) | 1406 | 164 | 79.37 | + | 2.07 | 1.89E-14 |
| cellular localization (GO:0051641) | 2174 | 252 | 122.73 | + | 2.05 | 2.43E-24 |
| positive regulation of cellular catabolic process (GO:0031331) | 416 | 48 | 23.48 | + | 2.04 | 3.62E-02 |
| RNA processing (GO:0006396) | 705 | 81 | 39.8 | + | 2.04 | 2.45E-05 |
| organophosphate biosynthetic process (GO:0090407) | 447 | 51 | 25.23 | + | 2.02 | 2.51E-02 |
| macromolecule localization (GO:0033036) | 2013 | 227 | 113.64 | + | 2 | 8.04E-20 |
| organic substance transport (GO:0071702) | 1899 | 211 | 107.2 | + | 1.97 | 2.50E-17 |
| secretion (GO:0046903) | 589 | 65 | 33.25 | + | 1.95 | 3.60E-03 |
| macromolecular complex subunit organization (GO:0043933) | 2074 | 228 | 117.08 | + | 1.95 | 1.52E-18 |
| cellular catabolic process (GO:0044248) | 1448 | 156 | 81.74 | + | 1.91 | 1.13E-10 |
| organic substance catabolic process (GO:1901575) | 1509 | 161 | 85.19 | + | 1.89 | 8.96E-11 |
| transmembrane receptor protein tyrosine kinase signaling pathway (GO:0007169) | 783 | 81 | 44.2 | + | 1.83 | 1.90E-03 |
| phosphorylation (GO:0016310) | 1269 | 130 | 71.64 | + | 1.81 | 6.20E-07 |
| single-organism biosynthetic process (GO:0044711) | 1333 | 136 | 75.25 | + | 1.81 | 2.82E-07 |
| macromolecular complex assembly (GO:0065003) | 1177 | 118 | 66.44 | + | 1.78 | 1.71E-05 |

| | | | | | | |
|---|---|---|---|---|---|---|
| single-organism transport (GO:0044765) | 3104 | 310 | 175.23 | + | 1.77 | 1.55E-20 |
| catabolic process (GO:0009056) | 1764 | 176 | 99.58 | + | 1.77 | 1.48E-09 |
| phosphate-containing compound metabolic process (GO:0006796) | 2020 | 201 | 114.03 | + | 1.76 | 2.47E-11 |
| single-organism localization (GO:1902578) | 3296 | 326 | 186.07 | + | 1.75 | 2.83E-21 |
| phosphorus metabolic process (GO:0006793) | 2067 | 204 | 116.69 | + | 1.75 | 3.27E-11 |
| cellular protein metabolic process (GO:0044267) | 3575 | 351 | 201.82 | + | 1.74 | 5.87E-23 |
| vesicle-mediated transport (GO:0016192) | 1150 | 112 | 64.92 | + | 1.73 | 2.04E-04 |
| carbohydrate derivative metabolic process (GO:1901135) | 1029 | 99 | 58.09 | + | 1.7 | 2.38E-03 |
| neuron development (GO:0048666) | 982 | 94 | 55.44 | + | 1.7 | 5.94E-03 |
| neuron projection development (GO:0031175) | 838 | 80 | 47.31 | + | 1.69 | 4.44E-02 |
| transport (GO:0006810) | 3742 | 357 | 211.24 | + | 1.69 | 3.11E-21 |
| establishment of localization (GO:0051234) | 3851 | 367 | 217.4 | + | 1.69 | 5.47E-22 |
| small molecule metabolic process (GO:0044281) | 2233 | 212 | 126.06 | + | 1.68 | 3.96E-10 |
| ion transport (GO:0006811) | 1263 | 114 | 71.3 | + | 1.6 | 6.55E-03 |
| protein metabolic process (GO:0019538) | 4241 | 380 | 239.41 | + | 1.59 | 3.93E-18 |
| localization (GO:0051179) | 4683 | 415 | 264.37 | + | 1.57 | 1.03E-19 |
| cellular component organization or biogenesis (GO:0071840) | 5188 | 455 | 292.87 | + | 1.55 | 1.09E-21 |
| organelle organization (GO:0006996) | 2912 | 255 | 164.39 | + | 1.55 | 4.28E-09 |
| cellular component biogenesis (GO:0044085) | 1978 | 173 | 111.66 | + | 1.55 | 5.82E-05 |
| cellular component organization (GO:0016043) | 5066 | 439 | 285.99 | + | 1.54 | 1.88E-19 |
| single-organism organelle organization (GO:1902589) | 1970 | 165 | 111.21 | + | 1.48 | 2.16E-03 |
| single-organism metabolic process (GO:0044710) | 4494 | 375 | 253.7 | + | 1.48 | 1.19E-12 |
| cellular nitrogen compound metabolic process (GO:0034641) | 5112 | 419 | 288.58 | + | 1.45 | 1.13E-13 |
| gene expression (GO:0010467) | 3825 | 307 | 215.93 | + | 1.42 | 2.75E-07 |
| nitrogen compound metabolic process (GO:0006807) | 5475 | 436 | 309.08 | + | 1.41 | 2.47E-12 |
| regulation of cellular component organization (GO:0051128) | 2160 | 172 | 121.94 | + | 1.41 | 2.52E-02 |
| regulation of cellular protein metabolic process (GO:0032268) | 2364 | 188 | 133.45 | + | 1.41 | 8.72E-03 |
| nervous system development (GO:0007399) | 2271 | 179 | 128.2 | + | 1.4 | 2.83E-02 |
| cellular metabolic process | 8573 | 669 | 483.97 | + | 1.38 | 1.01E-23 |

| | | | | | | |
|---|---|---|---|---|---|---|
| (GO:0044237) | | | | | | |
| regulation of protein metabolic process (GO:0051246) | 2530 | 195 | 142.82 | + | 1.37 | 3.91E-02 |
| cellular nitrogen compound biosynthetic process (GO:0044271) | 3407 | 261 | 192.33 | + | 1.36 | 9.48E-04 |
| nucleobase-containing compound metabolic process (GO:0006139) | 4372 | 331 | 246.81 | + | 1.34 | 2.80E-05 |
| primary metabolic process (GO:0044238) | 8697 | 657 | 490.97 | + | 1.34 | 1.12E-18 |
| cellular macromolecule metabolic process (GO:0044260) | 6753 | 507 | 381.22 | + | 1.33 | 9.07E-11 |
| heterocycle metabolic process (GO:0046483) | 4573 | 343 | 258.16 | + | 1.33 | 3.50E-05 |
| cellular aromatic compound metabolic process (GO:0006725) | 4575 | 342 | 258.27 | + | 1.32 | 5.42E-05 |
| metabolic process (GO:0008152) | 9928 | 742 | 560.46 | + | 1.32 | 1.06E-22 |
| regulation of biological quality (GO:0065008) | 3134 | 233 | 176.92 | + | 1.32 | 4.81E-02 |
| organic substance metabolic process (GO:0071704) | 9008 | 669 | 508.52 | + | 1.32 | 3.01E-17 |
| cellular biosynthetic process (GO:0044249) | 4493 | 331 | 253.64 | + | 1.3 | 4.96E-04 |
| organic cyclic compound metabolic process (GO:1901360) | 4818 | 354 | 271.99 | + | 1.3 | 1.72E-04 |
| organic substance biosynthetic process (GO:1901576) | 4587 | 335 | 258.95 | + | 1.29 | 9.53E-04 |
| biosynthetic process (GO:0009058) | 4673 | 340 | 263.8 | + | 1.29 | 1.06E-03 |
| macromolecule metabolic process (GO:0043170) | 7438 | 533 | 419.89 | + | 1.27 | 5.81E-08 |
| single-organism cellular process (GO:0044763) | 11415 | 777 | 644.4 | + | 1.21 | 1.82E-11 |
| cellular process (GO:0009987) | 14147 | 947 | 798.63 | + | 1.19 | 1.65E-18 |
| single-organism process (GO:0044699) | 12755 | 839 | 720.05 | + | 1.17 | 1.77E-09 |
| biological_process (GO:0008150) | 16542 | 1062 | 933.84 | + | 1.14 | 3.99E-20 |
| tissue development (GO:0009888) | 1601 | 52 | 90.38 | - | 0.58 | 3.21E-02 |
| G-protein coupled receptor signaling pathway (GO:0007186) | 1197 | 35 | 67.57 | - | 0.52 | 4.62E-02 |
| Unclassified (UNCLASSIFIED) | 4272 | 115 | 241.16 | - | 0.48 | 0.00E+00 |
| sensory perception (GO:0007600) | 945 | 24 | 53.35 | - | 0.45 | 2.88E-02 |
| anatomical structure formation involved in morphogenesis (GO:0048646) | 974 | 23 | 54.98 | - | 0.42 | 4.47E-03 |
| detection of chemical stimulus (GO:0009593) | 496 | 4 | 28 | - | < 0.2 | 1.24E-04 |

| | | | | | | |
|---|---|---|---|---|---|---|
| detection of stimulus involved in sensory perception (GO:0050906) | 514 | 3 | 29.02 | - | < 0.2 | 6.56E-06 |
| **PANTHER Protein Class** | **Homo sapiens - REFLIST (20814)** | **Client Text Box Input (1175)** | **Client Text Box Input (expected)** | **Client Text Box Input (over/under)** | **Client Text Box Input (fold Enrichment)** | **Client Text Box Input (P-value)** |
| SNARE protein (PC00034) | 42 | 14 | 2.37 | + | > 5 | 4.33E-05 |
| ribosomal protein (PC00202) | 225 | 73 | 12.7 | + | > 5 | 1.33E-29 |
| membrane traffic protein (PC00150) | 366 | 58 | 20.66 | + | 2.81 | 1.47E-09 |
| RNA binding protein (PC00031) | 859 | 133 | 48.49 | + | 2.74 | 1.15E-22 |
| membrane trafficking regulatory protein (PC00151) | 119 | 18 | 6.72 | + | 2.68 | 4.31E-02 |
| chaperone (PC00072) | 196 | 27 | 11.06 | + | 2.44 | 6.57E-03 |
| oxidoreductase (PC00176) | 609 | 62 | 34.38 | + | 1.8 | 2.07E-03 |
| nucleic acid binding (PC00171) | 2297 | 181 | 129.67 | + | 1.4 | 6.55E-04 |
| Unclassified (UNCLASSIFIED) | 9675 | 487 | 546.18 | - | 0.89 | 0.00E+00 |
| transcription factor (PC00218) | 1565 | 47 | 88.35 | - | 0.53 | 9.34E-05 |
| receptor (PC00197) | 1596 | 45 | 90.1 | - | 0.5 | 8.81E-06 |
| cell adhesion molecule (PC00069) | 507 | 11 | 28.62 | - | 0.38 | 2.74E-02 |
| G-protein coupled receptor (PC00021) | 468 | 9 | 26.42 | - | 0.34 | 1.52E-02 |
| defense/immunity protein (PC00090) | 551 | 8 | 31.11 | - | 0.26 | 1.44E-04 |
| cytokine receptor (PC00084) | 293 | 3 | 16.54 | - | < 0.2 | 1.12E-02 |
| cytokine (PC00083) | 196 | 1 | 11.06 | - | < 0.2 | 3.67E-02 |
| KRAB box transcription factor (PC00029) | 341 | 1 | 19.25 | - | < 0.2 | 1.55E-05 |

**C. Overrepresentation test GO-slim**

| PANTHER GO-Slim Molecular Function | Homo sapiens - REFLIST (20814) | Client Text Box Input (1175) | Client Text Box Input (expected) | Client Text Box Input (over/under) | Client Text Box Input (fold Enrichment) | Client Text Box Input (P-value) |
|---|---|---|---|---|---|---|
| structural constituent of ribosome (GO:0003735) | 180 | 57 | 10.16 | + | > 5 | 1.90E-22 |
| hydrogen ion transmembrane transporter activity (GO:0015078) | 41 | 10 | 2.31 | + | 4.32 | 2.50E-02 |
| translation initiation factor activity (GO:0003743) | 97 | 16 | 5.48 | + | 2.92 | 3.08E-02 |
| oxidoreductase activity (GO:0016491) | 626 | 65 | 35.34 | + | 1.84 | 5.71E-04 |
| RNA binding (GO:0003723) | 454 | 46 | 25.63 | + | 1.79 | 2.57E-02 |
| structural molecule activity (GO:0005198) | 1034 | 103 | 58.37 | + | 1.76 | 5.73E-06 |
| catalytic activity (GO:0003824) | 5209 | 373 | 294.06 | + | 1.27 | 2.19E-05 |

| | | | | | | |
|---|---|---|---|---|---|---|
| Unclassified (UNCLASSIFIED) | 10020 | 528 | 565.65 | - | 0.93 | 0.00E+00 |
| sequence-specific DNA binding transcription factor activity (GO:0003700) | 1462 | 44 | 82.53 | - | 0.53 | 2.05E-04 |
| DNA binding (GO:0003677) | 1878 | 53 | 106.02 | - | 0.5 | 4.18E-07 |
| nucleic acid binding transcription factor activity (GO:0001071) | 1646 | 44 | 92.92 | - | 0.47 | 6.85E-07 |
| receptor activity (GO:0004872) | 1636 | 41 | 92.36 | - | 0.44 | 7.91E-08 |
| serine-type peptidase activity (GO:0008236) | 322 | 4 | 18.18 | - | 0.22 | 1.13E-02 |
| cytokine activity (GO:0005125) | 230 | 1 | 12.98 | - | < 0.2 | 5.10E-03 |
| **PANTHER GO-Slim Biological Process** | **Homo sapiens - REFLIST (20814)** | **Client Text Box Input (1175)** | **Client Text Box Input (expected)** | **Client Text Box Input (over/under)** | **Client Text Box Input (fold Enrichment)** | **Client Text Box Input (P-value)** |
| oxidative phosphorylation (GO:0006119) | 56 | 15 | 3.16 | + | 4.74 | 2.65E-04 |
| translation (GO:0006412) | 435 | 90 | 24.56 | + | 3.66 | 8.77E-23 |
| protein localization (GO:0008104) | 116 | 19 | 6.55 | + | 2.9 | 1.15E-02 |
| respiratory electron transport chain (GO:0022904) | 217 | 33 | 12.25 | + | 2.69 | 1.28E-04 |
| generation of precursor metabolites and energy (GO:0006091) | 274 | 41 | 15.47 | + | 2.65 | 8.80E-06 |
| synaptic transmission (GO:0007268) | 331 | 36 | 18.69 | + | 1.93 | 4.70E-02 |
| organelle organization (GO:0006996) | 571 | 56 | 32.23 | + | 1.74 | 1.59E-02 |
| vesicle-mediated transport (GO:0016192) | 895 | 83 | 50.52 | + | 1.64 | 2.39E-03 |
| protein metabolic process (GO:0019538) | 2692 | 240 | 151.97 | + | 1.58 | 1.21E-10 |
| protein transport (GO:0015031) | 1082 | 91 | 61.08 | + | 1.49 | 3.03E-02 |
| cellular component organization or biogenesis (GO:0071840) | 1316 | 107 | 74.29 | + | 1.44 | 2.83E-02 |
| transport (GO:0006810) | 2473 | 184 | 139.61 | + | 1.32 | 1.54E-02 |
| metabolic process (GO:0008152) | 8247 | 572 | 465.56 | + | 1.23 | 4.33E-08 |
| primary metabolic process (GO:0044238) | 6825 | 463 | 385.29 | + | 1.2 | 2.48E-04 |
| Unclassified (UNCLASSIFIED) | 8629 | 409 | 487.13 | - | 0.84 | 0.00E+00 |
| developmental process (GO:0032502) | 2456 | 95 | 138.65 | - | 0.69 | 4.69E-03 |
| immune system process (GO:0002376) | 1391 | 44 | 78.53 | - | 0.56 | 1.99E-03 |
| transcription from RNA polymerase II promoter (GO:0006366) | 1723 | 54 | 97.27 | - | 0.56 | 1.12E-04 |

| | | | | | | |
|---|---|---|---|---|---|---|
| response to stimulus (GO:0050896) | 2170 | 68 | 122.5 | - | 0.56 | 2.95E-06 |
| transcription, DNA-dependent (GO:0006351) | 1941 | 60 | 109.57 | - | 0.55 | 1.12E-05 |
| regulation of transcription from RNA polymerase II promoter (GO:0006357) | 1319 | 40 | 74.46 | - | 0.54 | 1.13E-03 |
| regulation of nucleobase-containing compound metabolic process (GO:0019219) | 1700 | 50 | 95.97 | - | 0.52 | 1.48E-05 |
| mesoderm development (GO:0007498) | 671 | 17 | 37.88 | - | 0.45 | 2.16E-02 |
| ectoderm development (GO:0007398) | 663 | 15 | 37.43 | - | 0.4 | 4.83E-03 |
| sensory perception (GO:0007600) | 455 | 9 | 25.69 | - | 0.35 | 2.74E-02 |
| cell-cell adhesion (GO:0016337) | 391 | 7 | 22.07 | - | 0.32 | 3.75E-02 |
| skeletal system development (GO:0001501) | 232 | 1 | 13.1 | - | < 0.2 | 6.00E-03 |
