# Supplementary material for: When the brain goes diving: transcriptome analysis reveals a reduced aerobic energy metabolism and increased stress proteins in the seal brain
Source: BMC Genomics. 2016 Aug 9;17:583. doi: 10.1186/s12864-016-2892-y (PMC4979143; doi:10.1186/s12864-016-2892-y)
Supplement: Additional file 6: Figure S1. — Comparison of qRT-PCR and RNA-seq results. The changes in selected mRNA levels in visual cortices of the hooded seal and the ferret were estimated by qRT-PCR (black; n = 4) and RNA-seq (white). The mRNA levels of the genes S100B, Clu, SLC1A6 and GAPDH were evaluated. Both methods gave similar results. (PDF 22 kb) [file 12864_2016_2892_MOESM6_ESM.pdf]

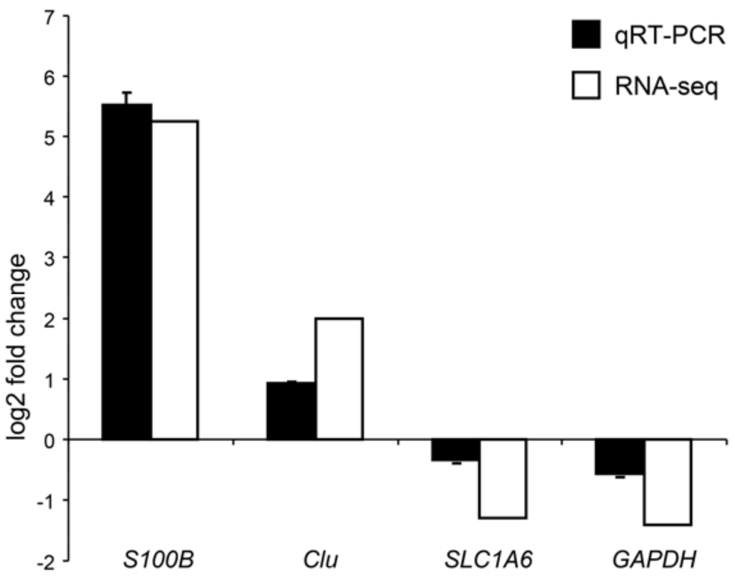

Additional File 6: Fig. S1. Comparison of qRT-PCR and RNA-seq results. The changes in selected mRNA levels in visual cortices of the hooded seal and the ferret were estimated by qRT-PCR (black; n = 4) and RNA-seq (white). The mRNA levels of the genes S100B, Clu, SLC1A6 and GAPDH were evaluated. Both methods gave similar results.
