# Supplementary material for: When the brain goes diving: transcriptome analysis reveals a reduced aerobic energy metabolism and increased stress proteins in the seal brain
Source: BMC Genomics. 2016 Aug 9;17:583. doi: 10.1186/s12864-016-2892-y (PMC4979143; doi:10.1186/s12864-016-2892-y)
Supplement: Additional file 8: Figure S2. — Correlation of gene expression between mammalian brain transcriptomes. The correlation coefficients were converted into distances and visualised by a neighbor-joining tree. (PDF 27 kb) [file 12864_2016_2892_MOESM8_ESM.pdf]

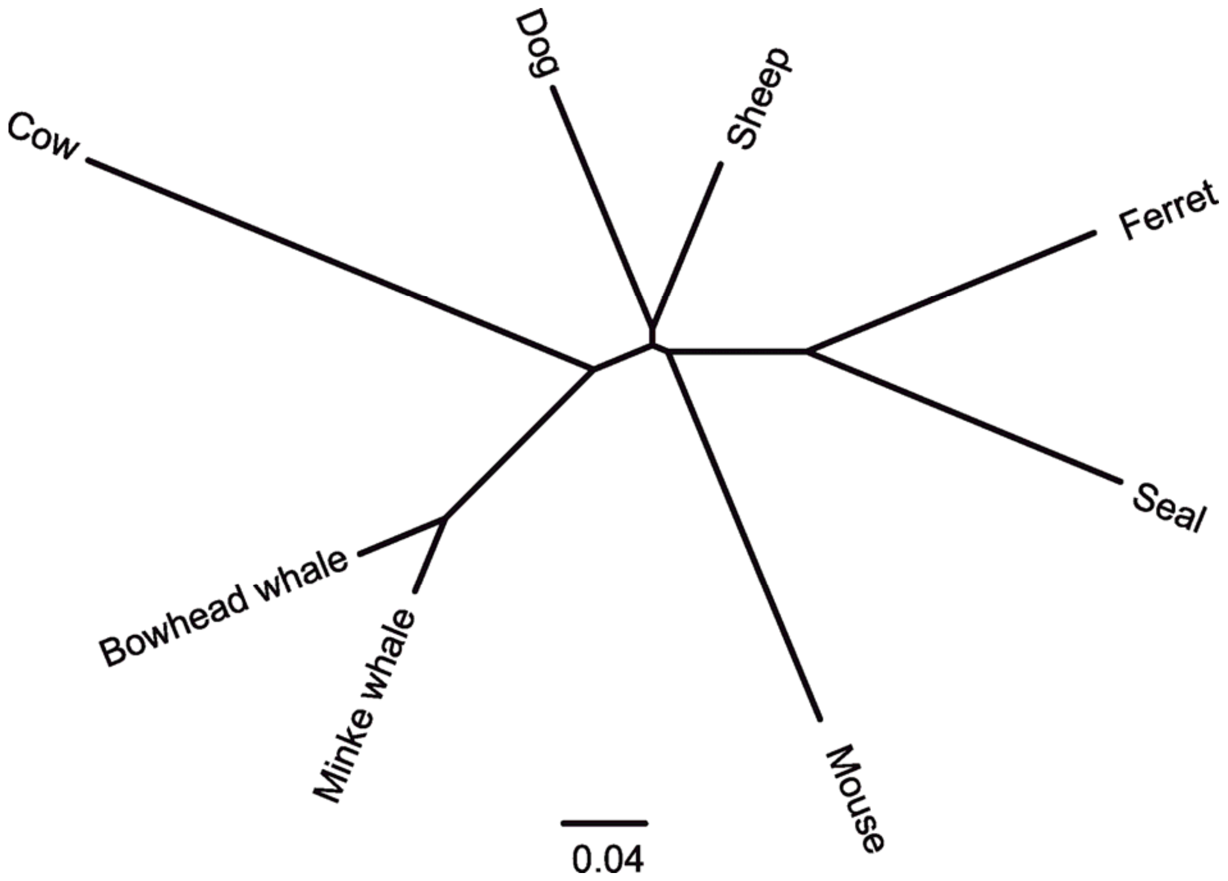

Additional File 8: Fig. S2. Correlation of gene expression between mammalian brain transcriptomes. The correlation coefficients were converted into distances and visualised by a neighbor-joining tree.
