# Supplementary material for: When the brain goes diving: transcriptome analysis reveals a reduced aerobic energy metabolism and increased stress proteins in the seal brain
Source: BMC Genomics. 2016 Aug 9;17:583. doi: 10.1186/s12864-016-2892-y (PMC4979143; doi:10.1186/s12864-016-2892-y)
Supplement: Additional file 9: Table S5. — List of primers. Forward and reverse primers, which had been generated according to the conserved sequences of the ferret and hooded seal genes, used in qRT-PCR expression analyses. (PDF 6 kb) [file 12864_2016_2892_MOESM9_ESM.pdf]

Additional File 9: Table S5. List of primers. Forward and reverse primers, which had been generated according to the conserved sequences of the ferret and hooded seal genes, used in qRT-PCR expression analyses.

**Primer**	**5'-3' sequence**
S100 forward	GATGAGCTCCTTGAGTTCGGA
S100 reverse	TGTCTGAGCTGGAGAAGGC
CLU forward	AGTTGTGACGGATCTCCTTGC
CLU reverse	GGCTCAGCAGGCCATGGAT
SLC1A6 forward	ATGCTGCAGATGCTGGTGCT
SLC1A6 reverse	CCGTGGTCACCATGTAGTAC
GAPDH forward	ACACGGAAGGCCATGCCAG
GAPDH reverse	CCTCTGGGAAGCTGTGGC
